# A Versatile Macroscale Platelet Membrane Coating: A Biomimetic Strategy to Balance Antithrombogenicity and Proendothelialization on Vascular Stents

**DOI:** 10.34133/research.1007

**Published:** 2025-12-12

**Authors:** Yongqi An, Cheng Ji, Hao Zhang, Hui Yan, Dimeng Wu, Rifang Luo, Yunbing Wang

**Affiliations:** ^1^ National Engineering Research Center for Biomaterials, Sichuan University, Chengdu 610064, China.; ^2^ Vanadium and Titanium Critical Strategic Materials Key Laboratory of Sichuan Province, Panzhihua University, Panzhihua 617000, China.; ^3^ Chengdu Minshan Institute of Biomaterials, Chengdu 610213, China.; ^4^ Research Unit of Minimally Invasive Treatment of Structural Heart Disease, Chinese Academy of Medical Sciences (No: 2021RU013), Beijing 100730, China.

## Abstract

Vascular stents require biointerfaces that simultaneously prevent thrombosis and promote endothelialization, yet achieving a balance of these conflicting demands on a single surface remains a challenge. While cell membrane coatings have demonstrated biomimetic potential, their application to macroscale devices has been largely limited to unstable noncovalent nanoparticle-focused approaches, which suffer from disordered assembly and poor stability under physiological flow. In contrast, we introduce a platelet membrane armor strategy facilitated by epigallocatechin gallate (EGCG) cross-linking, creating a robust macroscale coating that integrates both antithrombogenicity and proendothelialization. This covalent stabilization, achieved through EGCG’s unique chemistry, effectively anchors platelet membrane vesicles to form a stable coating that ensures uniform modification across various substrates. The coating is resistant to mechanical deformation and maintains its structural continuity even after over 30 d of rinsing. Importantly, the EGCG-cross-linked platelet membrane (EPM) coating preserves functional membrane proteins (CD47 and integrins). When stents coated with EPM armor were implanted in rabbits, they exhibited a marked reduction in acute thrombus formation, alongside enhanced endothelial cell proliferation and anti-inflammation effects, without introducing smooth muscle cell hyperplasia. This EPM coating strategy offers a promising approach to biomimetic interfaces for cardiovascular implants.

## Introduction

Drug-eluting stents effectively inhibit in-stent restenosis (ISR) by releasing antiproliferative drugs that suppress smooth muscle cell (SMC) proliferation, such as sirolimus, paclitaxel, and rapamycin [1]. However, these drugs also damage endothelial cells (ECs), delaying reendothelialization and thereby increasing the risk of late thrombosis [2,3]. In recent years, a growing number of researchers have devoted their efforts to developing drug-free functional coatings [4–6]. The aim is to achieve anticoagulant, anti-inflammatory, and antiproliferative effects through the coating, ultimately accelerating the formation of functional endothelial layers to protect the implanted vascular stents. In order to enhance the performance of coatings on vascular stents, numerous studies have been conducted with the aim of mimicking the natural endothelial environment [7,8]. This has led to the fabrication of a diverse range of functional stent coatings, including antifouling coatings with zwitterionic polymers [9–11], polysaccharide/protein coatings for anticoagulation [12–14], and nitric oxide (NO)-releasing coatings to facilitate endothelial regeneration [15–17]. Despite innovative research on drug-free functional coatings for stents, the implantation procedure inherently causes vascular endothelial injury; this damage, alongside the foreign body’s inflammatory and coagulation risks, often triggers exaggerated neointimal hyperplasia [18]. Nevertheless, balancing the inhibition of thrombosis and inflammation with the facilitation of endothelialization through material engineering remains challenging. Many modification techniques are prone to failure due to the adsorption of plasma proteins after implantation [19], which compromises their functionality—one of the key factors limiting the advancement of drug-free coatings.

To achieve a balanced interface with antifouling and proendothelialization properties, natural cell membranes present an ideal candidate. The cell membrane serves as a physiological barrier that mediates intercellular interactions [20]. Its phospholipid bilayer functions as an antifouling layer, protecting cells against harmful agents, while membrane proteins act as messengers, coordinating cell activities and facilitating intercellular signaling. The initial development of cell membrane coatings can be traced to the field of nanomedicine, where cell-membrane-coated nanoparticles have been shown to possess an extended in vivo circulation time, immune evasion, and specific targeting capabilities [21,22]. Although promising, these nanoparticle-focused approaches primarily result in fragile noncovalent coatings. Recent studies have demonstrated the potential for shifting the functional interface of cell membrane coatings from the nanoscale to the macroscale [23]. Benefiting from the unique properties of platelet membranes, we have previously designed a superhydrophilic surface based on cross-linked nanoparticle deposition. This interface spontaneously promote the adsorption and fusion of cell membrane vesicles at the interface, thus enabling the construction of large-scale cell membrane coatings [24]. However, the layers are primarily held together by weak noncovalent bonding forces, such as hydrophobic interactions and hydrogen bonding. This results in a lack of stability under flow conditions. Consequently, the preparation of the coatings frequently necessitates the use of high membrane concentrations or prolonged incubation periods, and there is a dearth of efficacious methodologies for assembling the coatings.

We hypothesize that the effective fabrication of macroscale cell membrane coatings relies on 3 key factors: efficient fusion between cell membrane vesicles, strong cohesion within the membrane coating, and robust adhesion of the membrane coating to the underlying substrate [25–27]. Intervening in these 3 elements is fundamental to achieving the high efficiency and stable functionality of macroscale cell membrane coatings. In this context, we are particularly excited about epigallocatechin gallate (EGCG), a natural polyphenol with notable biomaterial benefits. EGCG has been shown to facilitate rapid self-assembly into networks with cell membranes via noncovalent forces, including hydrogen bonds, ionic attractions, and hydrophobic interactions [28,29]. Moreover, the oxidized quinone structure of EGCG can react with nucleophilic compounds (sulfhydryl and amino groups) on the surface of cell membranes through the Michael addition/Schiff base reaction [30,31], further enhancing the stability of the coating.

**Fig. 1.**
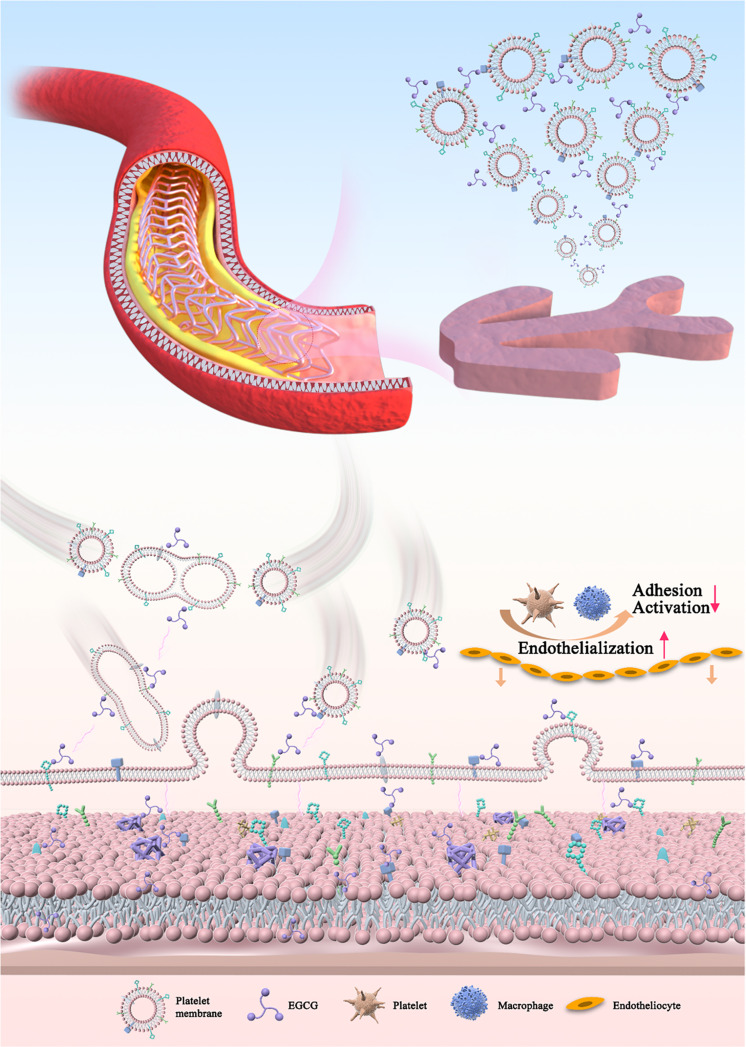
Schematic drawing of epigallocatechin gallate (ECGC)-cross-linked platelet membrane (EPM) coatings for vascular stents.

Based on the above considerations, we propose an EGCG-cross-linked platelet membrane (EPM) assembly strategy for the rapid modification and stable encapsulation of cell membrane coatings on macroscopic surfaces (Fig. 1). This method can be defined as a macroscale platelet membrane armor, a biomimetic coating designed to balance anticoagulation, anti-inflammation, and endothelialization on vascular stents. EPM can form robust, uniform, and stable macroscale coatings while preserving the native activity of membrane proteins. Such EPM coatings can serve as protective armor for vascular stents in vivo, endowing them with excellent antithrombogenicity and proendothelialization properties.

## Results and Discussion

### Design and construction of EPM coatings

The construction of a macroscale platelet membrane coating on materials that functions as a protective armor requires precise orchestration of interfacial interactions to balance structural integrity and bioactivity. In order to generate a uniform and firm cell membrane coating on the surface of irregular devices such as vascular stents, it relies on the abundance of functional groups on the surface of the cell membrane, such as hydroxyl, sulfhydryl, and amino groups [32]. Leveraging EGCG’s functionality as a mild cross-linker, we engineered a hybrid physicochemical strategy to stabilize platelet membrane vesicles (PMVs) while preserving their native protein architecture and tightly “locking” them together without compromising functionality.

Specifically, the Michael addition/Schiff base reactions between EGCG’s quinone groups (rapid formation during the oxidation of phenol groups) and membrane protein nucleophiles (–NH_2_ and –SH) are expected to enhance the cohesion of the membrane coating, without producing byproducts [30]. Moreover, EGCG can also act as a physical cross-linking agent to stabilize coatings through various intermolecular interactions (π–π stacking and enriched hydrogen bonding) in the coating [33]. We thus envision that EPM coatings—substrate independent, uniformly tunable, and highly stable—can armor vascular stents, imparting balanced antithrombogenicity and promoting endothelialization.

We began by depositing a thin polydopamine (PDA) layer onto the substrate’s surface. PDA coatings have been effectively employed and can be deposited onto diverse substrates—polymers, metals, or ceramics—thereby linking inorganic materials with organic reactions [34,35]. The reception and fusion of EGCG with cell membranes can be effectively achieved on the interface of PDA, benefiting from the abundant phenolic hydroxyl groups and hydrogen bonding sites on the PDA surface [36].

Building upon the 3 critical determinants of macroscale cell membrane coating fabrication—(a) efficient intervesicular fusion, (b) strong intracoating cohesion, and (c) robust substrate adhesion—we systematically investigated the interplay between EGCG cross-linker dosage and cell membrane vesicle concentration to optimize them for vascular stent applications. Comprehensive evaluation of coating morphology, platelet adhesion (Fig. S4), and quartz-crystal-microbalance-monitored assembly kinetics (Fig. S5) identified 0.5 mg/ml EGCG and 0.5 mg/ml platelet membrane protein final concentration as optimal parameters for fabricating EPM coatings. This protocol ensures homogeneous coating deposition with sustained stability while delivering reproducible antithrombogenic functionality critical for vascular device applications.

**Fig. 2.**
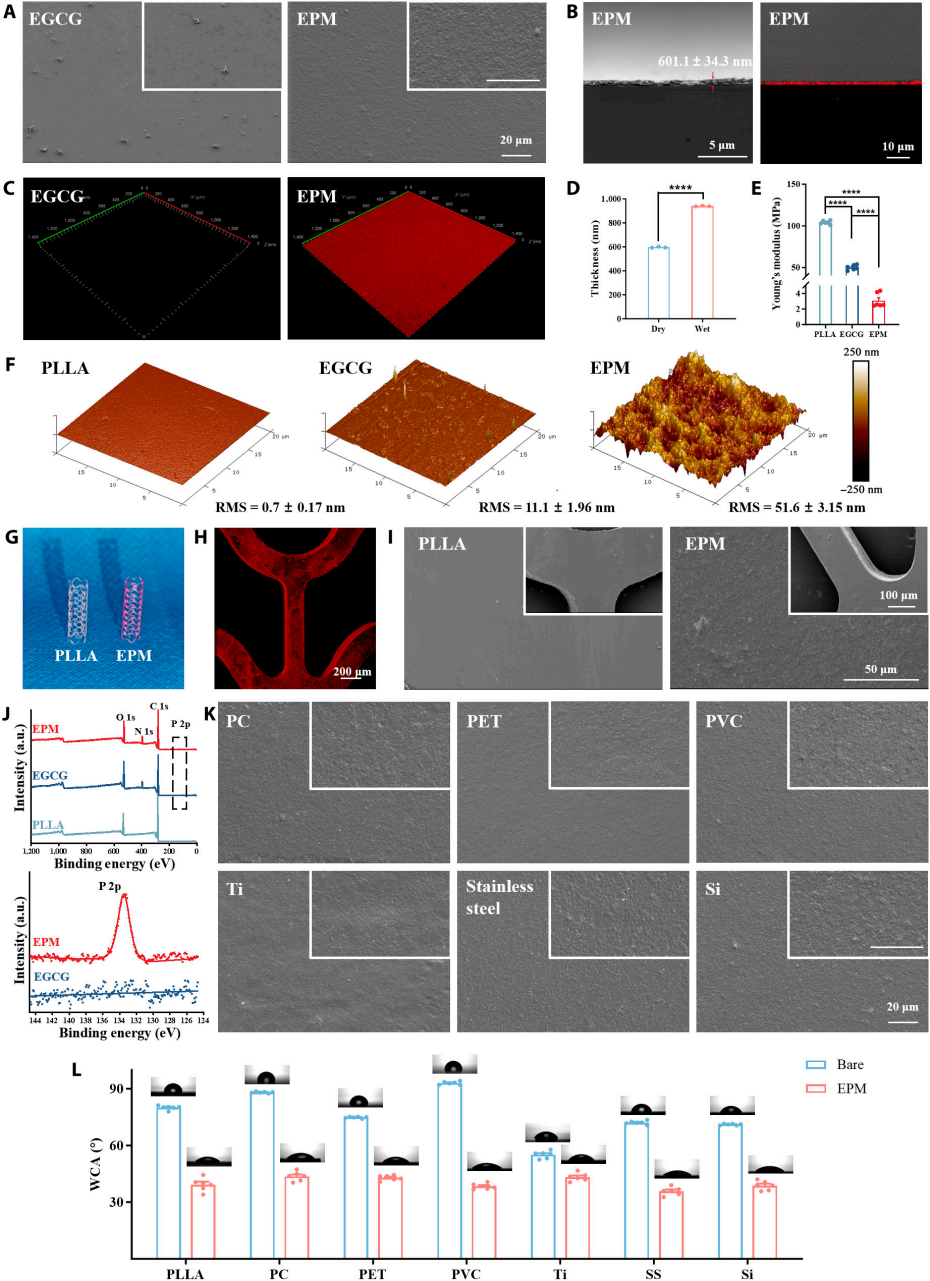
Development of EPM coatings for vascular stents. (A) Scanning electron microscopy (SEM) images of EGCG and EPM. (B) SEM images and fluorescence images of EPM cross-sections. (C) Fluorescence images labeled with rhodamine 6G. (D) The thickness of EPM coatings in the dry and wet states (*n* = 3). Quantification of the Young’s modulus of the samples in (E) wet states (*n* = 6). (F) Atomic force microscopy (AFM) images of sample surfaces in dry states. RMS, root mean square roughness. (G) Representative photographs of uncoated and EPM-coated poly-l-lactic acid (PLLA) stents, labeled with rhodamine 6G. (H) Fluorescence images labeled with rhodamine 6G of EPM-coated stents. (I) SEM images of uncoated and EPM-coated stents. (J) X-ray photoelectron spectroscopy (XPS) wide-scan spectrum and P 2p high-resolution narrow spectrum of PLLA, EGCG, and EPM. (K) SEM images of EPM coatings on different coated substrates. (L) Quantification of water contact angle before and after substrate coatings (SS for stainless steel, *n* = 6). PC, polycarbonate; PET, polyethylene terephthalate; PVC, polyvinyl chloride; WCA, water contact angle.

Scanning electron microscopy (SEM) images (Fig. 2A) showed that the EGCG coating contained dispersed oxide particles—originating from dopamine’s self-polymerization into PDA particles and their subsequent reaction with added EGCG—whereas the EPM coating showing a more homogeneous, but undulating, continuous planar morphology. Confocal laser scanning microscopy (CLSM) of rhodamine 6G-labeled coatings confirmed the retention and homogeneity of platelet membranes across substrates, with cross-sectional imaging revealing a uniform EPM nanolayer measuring 601.1 ± 34.3 nm thick (Fig. 2B and C). The thickness of the EPM coatings was 596.6 ± 4.2 and 939.8 ± 3.7 nm, respectively, in dry and wet states (Fig. 2D). Substrate stiffness mediates cellular responses in representative tissues, which initiate intracellular signaling cascades that ultimately lead to cellular outcomes such as migration, proliferation, and differentiation [37]. As shown in Fig. 2E, the Young’s moduli of the poly-l-lactic acid (PLLA), EGCG, and EPM samples were 104.1 ± 2.1, 50.0 ± 3.0, and 3.1 ± 0.9 MPa, respectively. Atomic force microscopy quantified the surface nanotopography, showing controlled roughness arising from precisely assembled PMVs compared to relatively smooth PLLA and EGCG surfaces (Fig. 2F).

As shown in Fig. 2G, direct observation of uncoated and EPM-coated PLLA stents labeled with rhodamine 6G revealed that the EPM-coated stents showed a more pronounced red fluorescence. Furthermore, the CLSM image showed a uniform EPM coating on the surface of the stents (Fig. 2H). The uncoated and EPM-coated stents were observed by SEM (Fig. 2I). While the PLLA surface was relatively smooth, the EPM coating exhibited a rougher surface, with retention and uniformity similar to that on planar substrates, confirming the successful coating of EPM on the stents’ nonplanar surface. To further evaluate the mechanical stability of the EPM coating, EPM stents were dilated with an angioplasty balloon in phosphate-buffered saline (PBS) (Fig. S6). The EPM coating demonstrated good stability after implantation and mechanical deformation, exhibiting some cracks after balloon dilation but overall remaining intact.

X-ray photoelectron spectroscopy (Fig. 2J) was employed to analyze elemental distributions on the surfaces of the uncoated and EGCG- and EPM-coated PLLA sheets. The EGCG coating is a very thin layer, and it is the nitrogen signals in the substrate PDA that can be detected. Specific peaks indicate the presence of nitrogen (N 1s) and phosphorus (P 2p) in the EPM coatings, and the presence of biofilm-specific elemental peaks further indicates platelet membrane coverage [38].

Due to the versatility of EGCG in directing uniform priming formation on diverse materials, EPM coatings are considered to have the potential to be prepared on several substrate surfaces, including polymer substrates (polycarbonate [PC], polyethylene terephthalate [PET], and polyvinyl chloride [PVC]), metal substrates (316L stainless steel and Ti), and silicon wafers using the same coating protocol. The coating morphologies were examined using SEM (Fig. 2K), all of which showed uniform and continuous platelet coating morphologies. Surface energy characterization via water contact angles showed consistent hydrophilic behavior (~40°) across all coated materials (Fig. 2L), confirming the integrity of the biomimetic layer regardless of the underlying substrate chemistry.

### Retention of platelet membrane proteins on EPM and the coatings’ stability

**Fig. 3.**
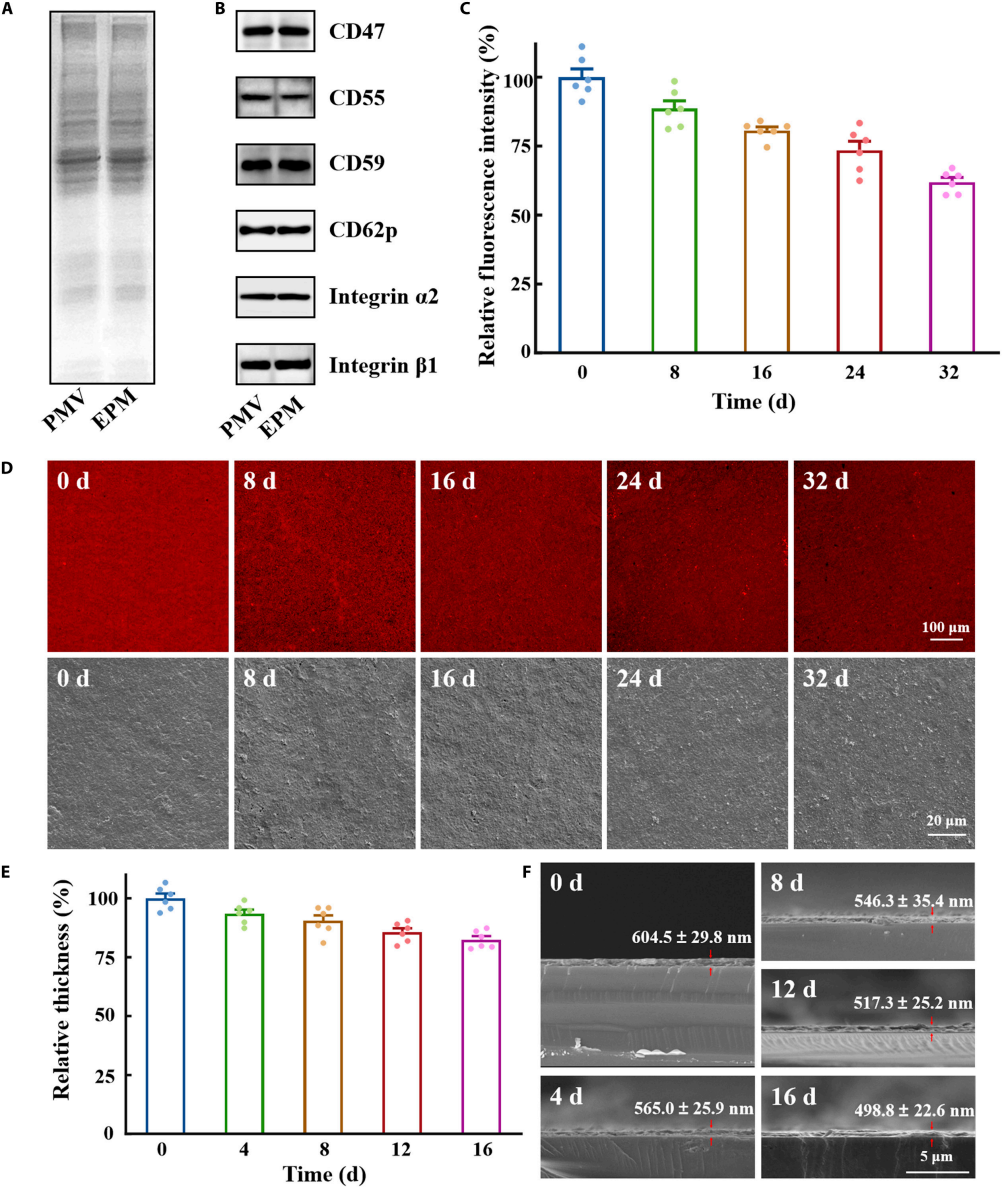
Retention of EPM coatings. (A) Sodium dodecyl sulfate–polyacrylamide gel electrophoresis (SDS-PAGE) analysis of proteins in platelet membrane vesicles (PMVs) and EPM. (B) Western blot analysis of platelet membrane proteins in PMVs and EPM. Stability of EPM coating in a simulated flow environment. (C) Fluorescence intensity of EPM coating residues (*n* = 6). (D) Fluorescence images and SEM images of the residual coating after 8, 16, 24, and 32 d of rinsing. Scale bars, 100 and 20 μm. (E) Thickness of EPM coating residues (*n* = 6). (F) SEM images of the residual coating cross-sections after 4, 8, 12, and 16 d of rinsing. Scale bar, 5 μm.

To verify the retention of platelet membrane proteins on EPM, sodium dodecyl sulfate–polyacrylamide gel electrophoresis (SDS-PAGE) analysis was performed (Fig. 3A). The global banding pattern of EPM closely resembled that of PMV, indicating successful preservation of key protein components throughout the coating process. Western blot analysis (Fig. 3B) further confirmed the retention of hallmark immune-evasion proteins—including CD47, CD55, and CD59—along with adhesion-related markers such as integrins α2, β1, and CD62P. These results collectively demonstrate that essential membrane characteristics remain detectable following EPM formation. Mechanistically, CD47 binding to SIRPα on phagocytes transmits a “don’t-eat-me” signal [39–41], while CD55 and CD59 jointly restrict complement amplification and membrane attack complex assembly. Together, these pathways help minimize opsonization and phagocytic clearance. Supporting evidence from membrane-coated nanoplatforms indicates that platelet membrane cloaking reduces macrophage uptake and suppresses complement activation in human plasma, contingent on the surface retention of CD47, CD55, and CD59 [42]. Moreover, studies involving red blood cell–membrane systems have shown that genetic ablation or functional loss of CD47 shortens circulation half-life and accelerates clearance, underscoring its critical role in sustaining in vivo persistence [43,44]. Similarly, the complement-regulatory functions of CD55 and CD59 have been validated through antibody-blocking experiments on membrane vesicles and tumor cells, where neutralization of either protein elevates C3 deposition and membrane attack complex formation, thereby enhancing complement-dependent cytotoxicity [45,46]. Although adhesion proteins such as integrins and CD62P can facilitate binding in particle-based delivery systems [47,48], their functional impact within an immobilized stent coating is likely constrained by suboptimal orientation and the absence of active mechanotransduction. Thus, we interpret their detectability primarily as evidence of structural preservation rather than functional engagement under these conditions. Taken together, the consistent proteomic profile between EPM and PMV, combined with targeted validation of key immunomodulatory proteins, supports the conclusion that EPM retains biologically relevant platelet membrane interfaces conducive to hemocompatibility. These features are anticipated to mitigate thromboinflammatory activation and support endothelialization on blood-contacting implants in vivo. Although comprehensive profiling of all membrane proteins remains impractical, the orthogonal assays employed here provide converging evidence that EPM preserves fundamental functional determinants of the native platelet membrane suitable for in vivo application.

Coating stability under physiological shear stress was assessed via rhodamine 6G staining for quantitative analysis. EPM maintained 88.7% ± 7.0% coverage at day 8—a critical window for acute postimplantation responses [49]—with gradual erosion to 61.8% ± 4.8% by day 32 (Fig. 3C). While this progressive reduction indicates surface erosion phenomena, SEM/CLSM morphology (Fig. 3D) analysis confirmed preserved structural continuity across the substrate interface. Cross-sectional SEM images (Fig. 3E and F) further revealed that after 8 d of rinsing in a mobile phase, the coating thickness remained at 90.4% ± 5.8% of its initial value, and after 16 d, it still retained 82.5% ± 3.7% of the original thickness. This retention can be attributed to the covalently cross-linked networks facilitated by EGCG, which effectively resist delamination. Owing to its robust nature, the EPM coating process has been shown to successfully bind and retain functional proteins on platelet membranes and exhibits reliable stability over a period of time, a key property for its potential use as a protective armor for cardiovascular implants.

### Hemocompatibility of the coatings

**Fig. 4.**
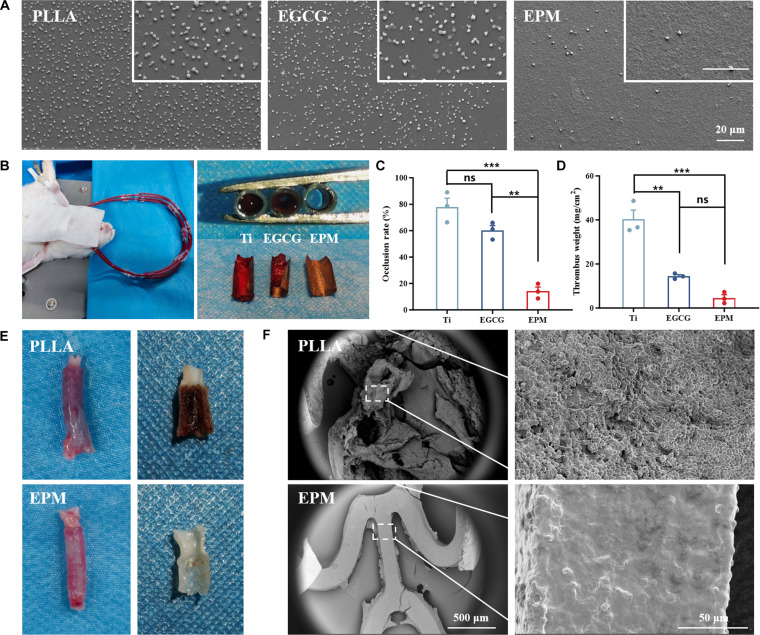
Effects of EPM coatings on hemocompatibility. (A) Morphology of adhered platelets on sample surfaces. (B) The digital photographs of ex vivo circulation thrombogenicity model of rabbits and thrombi deposited on uncoated, EGCG, and EPM Ti samples (*n* = 3). Quantitative analysis of (C) occlusion rate and (D) thrombus weight. The digital photographs (E) and SEM images (F) of acute thrombosis assay of implanted uncoated and EPM stents.

Since biomedical devices come into contact with blood after implantation, it is crucial to assess their antithrombotic performance both in vitro and in vivo. Platelet adhesion and activation on foreign surfaces usually trigger blood coagulation [50]. The anticoagulant capacity of the coating was assessed by an in vitro platelet adhesion test (Fig. 4A). After 1 h of incubation, extensive platelet adhesion and spreading with dendritic morphology were observed on PLLA and EGCG surfaces, indicative of an activated state that may lead to thrombus formation [51]. In contrast, the EPM coating demonstrated notably reduced platelet adhesion and no activated platelets, attributed to the inherent antifouling properties of its phospholipid bilayer, suggesting a beneficial role in thrombosis prevention.

To assess antithrombogenicity under physiological flow, an ex vivo circulation test was carried out in New Zealand white rabbits by shunting blood from the carotid artery to the jugular vein through coated titanium samples (Fig. 4B). The coated titanium samples were used as test samples. After 1 h of ex vivo circulation, the circuits were disassembled and directly observed, and abundant thrombi appeared on the surface of the untreated titanium samples, which had nearly blocked the access, and an obvious thrombus was also observed on the EGCG-coated titanium samples, whereas occasional thrombus attachment was visible on the EPM-coated titanium samples. The occlusion rate of the circuit (Fig. 4C) and the weight of thrombus per unit area of the sample surface (Fig. 4D) were then quantitatively assessed. The occlusion rates of bare Ti and EGCG-coated samples were about 78% and 60%, respectively. Notably, the occlusion rate of the EPM-coated samples was dramatically reduced to 14%. The weight of the thrombus adhering to the EPM surface was significantly reduced compared to those for the bare Ti group.

We further evaluated the in vivo thrombogenic ability of the coatings by implanting the stent in a rabbit’s abdominal aorta for acute thrombosis test. After stent implantation for 6 h, the blood vessels containing stents were harvested, and a severe thrombus was observed on the surface of the PLLA stent (Fig. 4E), and from the SEM image after dehydration, severe thrombi were deposited on the PLLA stent (Fig. 4F). However, the EPM-coated PLLA stent was quite clean with no obvious thrombus deposition, which was consistent with the in vitro and ex vivo hemocompatibility test shown above. These results demonstrate that the EPM coating acts as an effective armor, conferring excellent anticoagulant properties. This effect stems from the dual protective mechanisms of the platelet membrane—an antifouling phospholipid layer combined with membrane proteins that provide immune camouflage. These mechanisms enable platelet-membrane-coated nanoparticles to circulate safely in the bloodstream without inducing thrombotic risk [42,52,53]. Leveraging these same features, the macroscopic EPM coating also imparts outstanding antithrombotic performance to medical devices, substantially enhancing their hemocompatibility.

### Cytocompatibility of the coatings

Vascular stent implantation inevitably destroys the vessel wall during implantation. Incomplete healing of ECs leads to late-stent thrombosis, while overproliferation of SMCs leads to severe intimal hyperplasia, which may result in ISR [54]. Following tissue damage, macrophages congregate on and adhere to the stent surface, where even subtle microenvironmental changes robustly drive their polarization into M1 or M2 phenotypes [55]. To assess the cytocompatibility of the coating, human umbilical vein ECs, human umbilical artery SMCs, and macrophage cell line RAW264.7 were cultured on the samples.

**Fig. 5.**
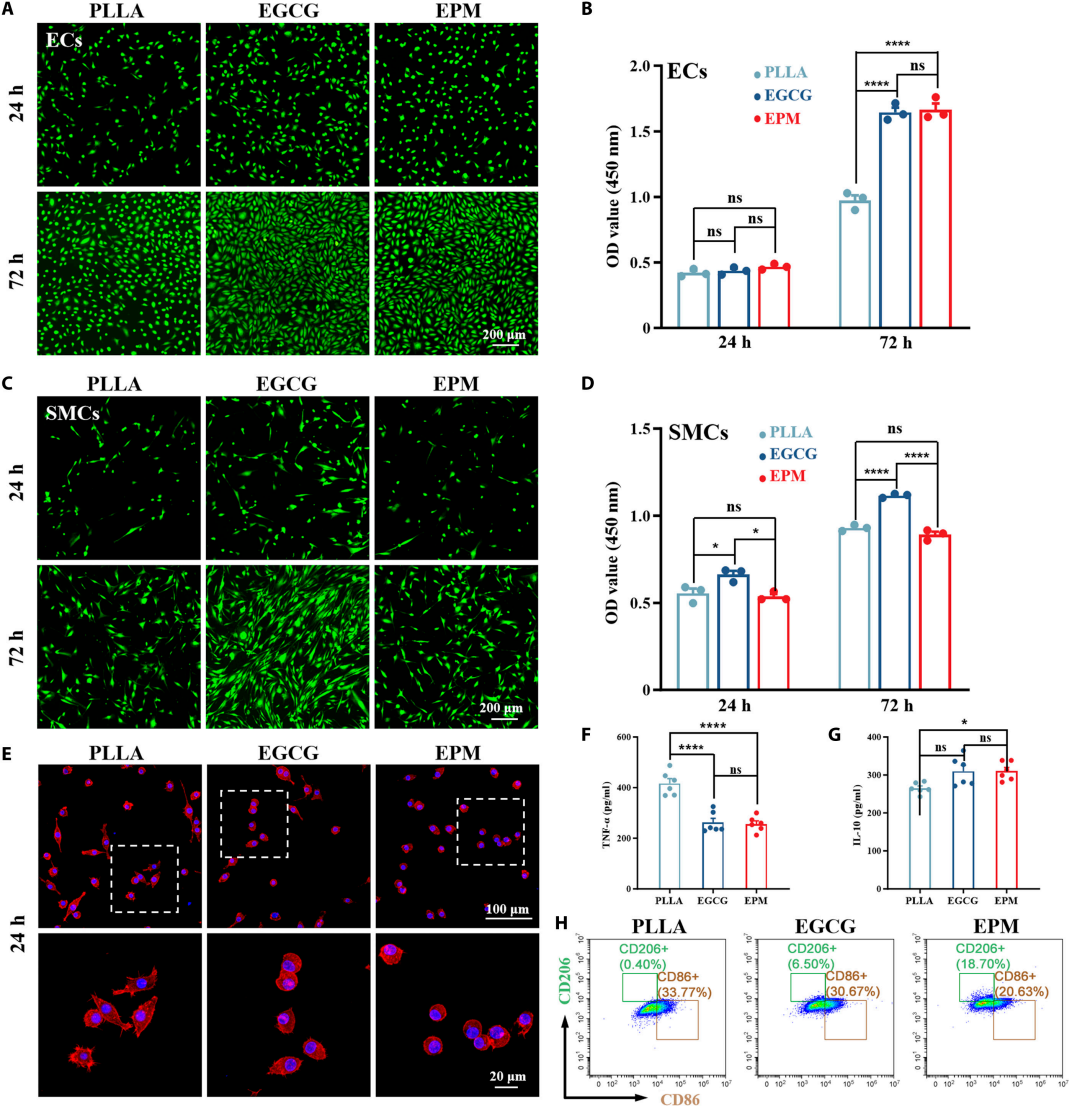
Cytocompatibility and anti-inflammatory capacity of EPM coatings. Fluorescence images of (A) endothelial cells (ECs) and (C) smooth muscle cells (SMCs) cultured on samples for 24 and 72 h. Live cells were stained with fluorescein diacetate (green). Quantitative analysis of the cell viability of (B) ECs and (D) SMCs cultured on samples for 24 and 72 h (*n* = 6). OD, optical density. (E) Fluorescence images of RAW264.7. F-actin was labeled with tetramethylrhodamine isothiocyanate-conjugated phalloidin (red), and nuclei were counterstained with 4′,6-diamidino-2-phenylindole (DAPI; blue). Expression levels of (F) tumor necrosis factor-α (TNF-α) and (G) interleukin-10 (IL-10) of the culture supernatant of RAW264.7 cells, measured using an enzyme-linked immunosorbent assay (ELISA kit) (*n* = 6). (H) Flow cytometry results of macrophage polarization after culture of different samples.

EGCG was selected as the cross-linking agent due to its mild reaction properties and demonstrated cytocompatibility, which provides a supportive microenvironment for cellular proliferation. As shown in Fig. 5A, there was no appreciable difference in the number of ECs adhering to EGCG and EPM after 24 and 72 h of incubation, both of which were considerably higher than that of PLLA, suggesting that EPM has the ability to support the growth of ECs. The Cell Counting Kit-8 (CCK-8) results showed (Fig. 5B) that the cell viability of adherent ECs on EGCG and EPM was significantly better than that of the control, whereas there was no significant difference between EGCG and EPM. The morphology of fluorescence-stained SMCs was observed after 24 and 72 h of culture (Fig. 5C), and SMCs adhering to EGCG showed a distinct spindle-like morphology. The cell viability of SMCs adhering to EPM was considerably lower compared with that for EGCG, while it showed no clear difference relative to that for PLLA. The CCK-8 results (Fig. 5D) showed that the cell viability of SMCs adhering to EGCG was better than that for the control and EPM, while there was no significant difference between the latter 2 groups. With increasing substrate stiffness, SMCs underwent a phenotypic switch from a contractile to a synthetic state, driving accelerated SMC proliferation in the EGCG-treated group [56]. This indicates that the EPM coating preserves a rich complement of membrane proteins, which in turn enhances EC proliferation and migration. Moreover, the resulting soft layer (retaining natural membrane flexibility) does not promote the rapid growth of SMCs, a feature that is advantageous for preventing restenosis [57].

The inflammatory response was evaluated by assessing RAW264.7 cell adhesion and phenotype. Early inflammatory activation following biomaterial implantation is a critical determinant of immune rejection [58]. Hence, examining macrophage behavior on material surfaces serves as a predictive measure of the in vivo inflammatory response. Previous studies have established a strong correlation between macrophage morphology and polarization [59]. CLSM observations (Fig. 5E) after 24 h of culture revealed that macrophages adhered to the sample surfaces. On the PLLA surfaces, cells exhibited notable elongation and stretching—morphological changes typically associated with an inflammatory response.

In contrast, while EGCG samples showed similar morphological alterations, EPM surfaces predominantly hosted more rounded macrophages, indicating a potential in vitro anti-inflammatory effect. Enzyme-linked immunosorbent assay (ELISA) results (Fig. 5F and G) confirmed that the EGCG and EPM groups had significantly lower levels of the pro-inflammatory cytokine tumor necrosis factor-α (TNF-α) compared to the PLLA group, while the anti-inflammatory cytokine interleukin-10 (IL-10) was relatively elevated.

Furthermore, macrophage polarization was assessed by flow cytometry using CD206 and CD86 as surface markers for the M2 and M1 phenotypes, respectively [60]. Classically activated M1 macrophages are generally associated with pro-inflammatory responses, whereas M2 macrophages exhibit anti-inflammatory properties. Platelet-membrane-coated nanoparticles have also been shown to enhance the immunomodulatory function of nanoparticles [61,62]. As shown in Fig. 5H, compared with the PLLA and EGCG groups, the EPM treatment markedly promoted M2 polarization while concurrently suppressing M1 polarization. This shift was specifically demonstrated by the decreased CD86 expression in M1 macrophages and the increased CD206 expression in M2 macrophages.

In summary, these results suggest that EPM coatings provide a favorable microenvironment for vascular endothelialization and may contribute to the reduction of inflammatory responses, thus supporting their use in vascular stents.

### Tissue compatibility of the coatings in rats

**Fig. 6.**
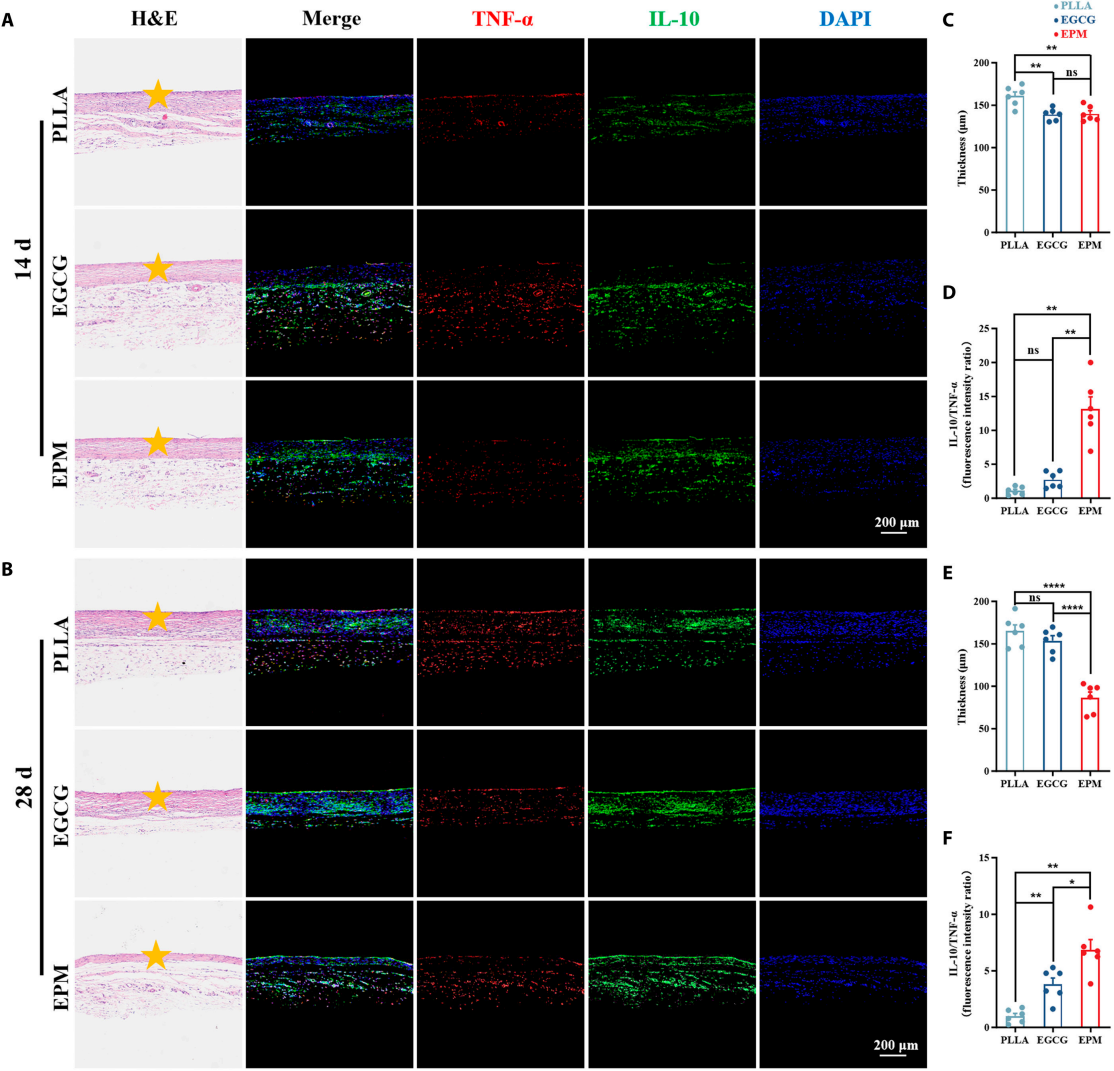
Effects of EPM coatings on inflammatory response in rats. Subcutaneously implanted sample stained by hematoxylin and eosin (H&E), TNF-α, and IL-10 for (A) 14 and (B) 28 d. The PLLA implants are indicated by orange stars in the histological sections. The thickness of the surrounding fibrous capsules for each group was quantified from the corresponding H&E-stained images for (C) 14 and (E) 28 d (*n* = 6). Quantification of the IL-10/TNF-α ratio for (D) 14 and (F) 28 d (*n* = 6).

To further verify the immune response of the samples, we performed subcutaneous implantation experiments in Sprague–Dawley (SD) rats. After implanting the samples subcutaneously in SD rats for 14 and 28 d, the samples wrapped with surrounding tissues were collected for hematoxylin and eosin (H&E) immunohistochemical staining, and TNF-α/IL-10 immunofluorescence staining (Fig. 6A and B). The implant’s inflammatory response was assessed by the thickness of the fibrous capsule and the relative levels of cytokines. Inflammatory cell infiltration was increased, and a thicker fibrous envelope indicated a more severe tissue reaction [63]. The IL-10/TNF-α ratio was also used as a marker of the inflammatory state, with higher values indicating less inflammation and possibly a post-inflammatory repair phase [64]. The average thickness of the fibrous capsule was determined; a quantitative analysis of the relative intensity of IL-10/TNF-α expression was also conducted (Fig. 6C to F). Quantitative analysis showed that at 14 d, the fiber thickness was 160.78 ± 11.92 μm in the PLLA group and was reduced in both the EPM (139.76 ± 8.90 μm) and EGCG groups (139.12 ± 6.99 μm), but there was no significant difference between the 2 groups. At 28 d, the fiber thickness was significantly reduced in the EPM group; the fiber thickness was 153.68 ± 14.50 μm for EGCG and increased (165.04 ± 17.92 μm) for PLLA compared to that for EPM (86.18 ± 16.99 μm). From the H&E results, we found that the fiber capsule formed around the PLLA sheet was significantly thicker than that of the EPM group at the indicated time points. Immunofluorescence staining results showed that the EPM-modified group had higher values of IL-10/TNF-α staining results on day 14 and day 28, and the tissues were in a reparative state with a weak inflammatory response compared with the PLLA group.

Compared with those for PLLA and EGCG, the fibrous capsule on the EPM surface was thinner and the relative intensity of inflammation-related protein expression was lower. According to this result, EPM-induced inflammatory stimulation is temporary, and with prolonged placement time, the membrane proteins on the surface and its own cell membrane components act to attenuate the inflammatory response. The anti-inflammatory properties of EPM likely arise not only from the inherent biocompatibility of its phospholipid-bilayer surface but also from the platelet membrane’s immune escape proteins [65]. The EPM coating was confirmed to be effective in reducing the in vivo inflammatory response to PLLA substrates with good histocompatibility.

### Stent implantation in rabbits

**Fig. 7.**
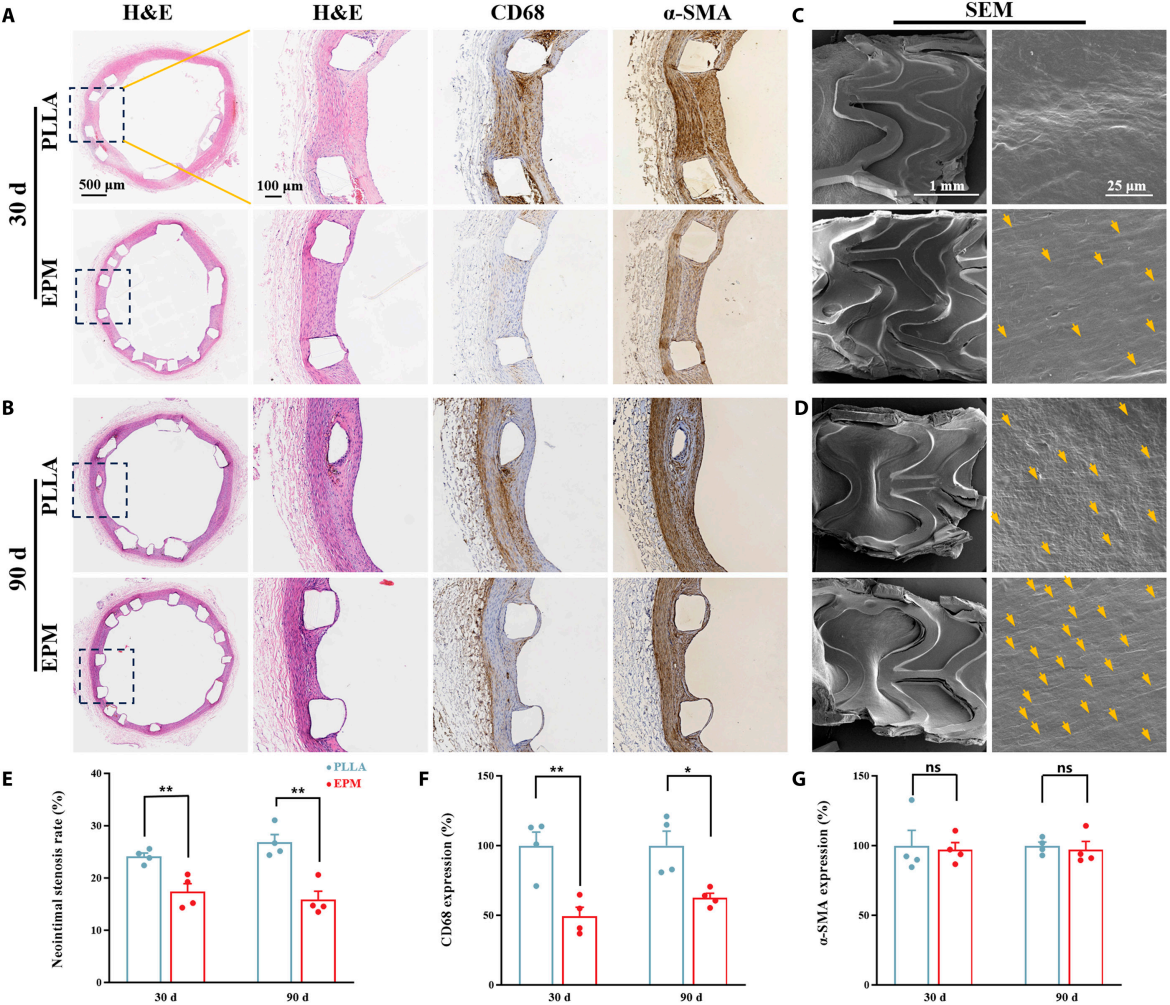
Vascular stent deployment in rabbits. Vascular tissues collected at (A) 30 and (B) 90 d postimplantation were stained with H&E, CD68, and α-smooth muscle actin (α-SMA). SEM micrographs of the luminal surfaces at (C) 30 and (D) 90 d postimplantation show yellow arrows denoting representative cell growth. Quantitative analyses include (E) neointimal stenosis ratio, (F) CD68-positive area, and (G) α-SMA-positive area in stented vessels (*n* = 4).

To further investigate whether the EPM armor can effectively facilitate in situ endothelialization while suppressing neointimal hyperplasia, we performed in vivo stent implantation experiments in a rabbit model for 30 and 90 d, respectively. After the predetermined time, the stents embedded in blood vessels were harvested and the tissues were further treated with H&E and immunohistochemical staining to verify the proliferation and inflammatory response (Fig. 7A and B and Fig. S7a and b).

SEM images showed that at different time points of 30 (Fig. 7C) and 90 d (Fig. 7D), on the luminal surface of neovascular vessels, cells on the EPM-coated stent surface grew more along the direction of blood flow, while cells on the bare stent surface exhibited a disordered arrangement. This indicates an improvement in the performance of the EPM-coated stent. At 90 d, a uniformly organized layer of cells fully enveloped the struts of the EPM-modified PLLA stents, growing in a spindle-like shape and with a high degree of orientation, a typical EC morphology.

Quantitative analysis (Fig. 7E) showed that the intima formed around the bare PLLA stent was thicker than that formed around the EPM stent. Neointimal hyperplasia was most severe in the bare PLLA stent group, with 30- and 90-d luminal stenosis rates of 24.11% ± 1.34% and 26.80% ± 2.98%, respectively. The 30- and 90-d restenosis rates were reduced to 17.36% ± 3.07% and 15.84% ± 3.20% for the EPM group, respectively, suggesting stronger inhibition of neointimal hyperplasia.

To assess the inflammatory response, tissue sections were stained for CD68, CD86, and CD206 for semiquantitative scoring of the degree of inflammation, pro-inflammatory, and anti-inflammatory activity. Meanwhile, the proliferation of vascular SMCs was analyzed semiquantitatively by the phenotypic recognition marker α-smooth muscle actin (α-SMA), and the expression intensity of the bare PLLA stent was set at 100%.

As illustrated in Fig. 7F, the CD68 expression levels of the EPM-modified stents were significantly lower than those of the PLLA stents after 30 and 90 d of implantation. The EPM group exhibited a notable anti-inflammatory capacity, with expression levels of 49.32% ± 12.85% and 62.58% ± 6.40%, respectively. To further elucidate the differences in inflammatory regulation between these 2 groups, immunohistochemical analyses of CD86 and CD206 were performed (Fig. S7c and d). The results demonstrated that the expression level of the M1 macrophage marker CD86 was significantly lower than that in the PLLA group, with 39.04% ± 7.11% and 37.29% ± 7.68% at 30 and 90 d, respectively. Conversely, despite a reduction in the total macrophage CD68 expression, the level of the M2 macrophage marker CD206 was elevated in the EPM group, with 85.49% ± 7.12% and 78.82% ± 8.28%, respectively. This was a notable contrast to the PLLA group. As illustrated in Fig. 7G, the α-SMA expression level on the surface of the EPM-coated stents was marginally higher than that of the PLLA stents at 30 and 90 d of implantation, although the difference was not statistically significant. The in vitro macrophage flow cytometry results collectively demonstrate that even in the absence of pharmaceutical agents typically used in drug-eluting stents, the EPM coating promotes macrophage polarization toward the anti-inflammatory and pro-reparative M2 phenotype, thereby favoring vascular neointimal remodeling.

The EC layer lining the vessel wall plays a crucial role in maintaining the vascular microenvironment. Healthy endothelial membranes are rich in natural anticoagulants, such as heparan, prostaglandins, and NO [66]. They produce and release a variety of bioactive factors that modulate vessel constriction and dilation, thereby maintaining proper blood flow and long-term patency. Consequently, promoting rapid endothelial coverage of implanted vascular stents is a central goal in stent surface modification.

**Fig. 8.**
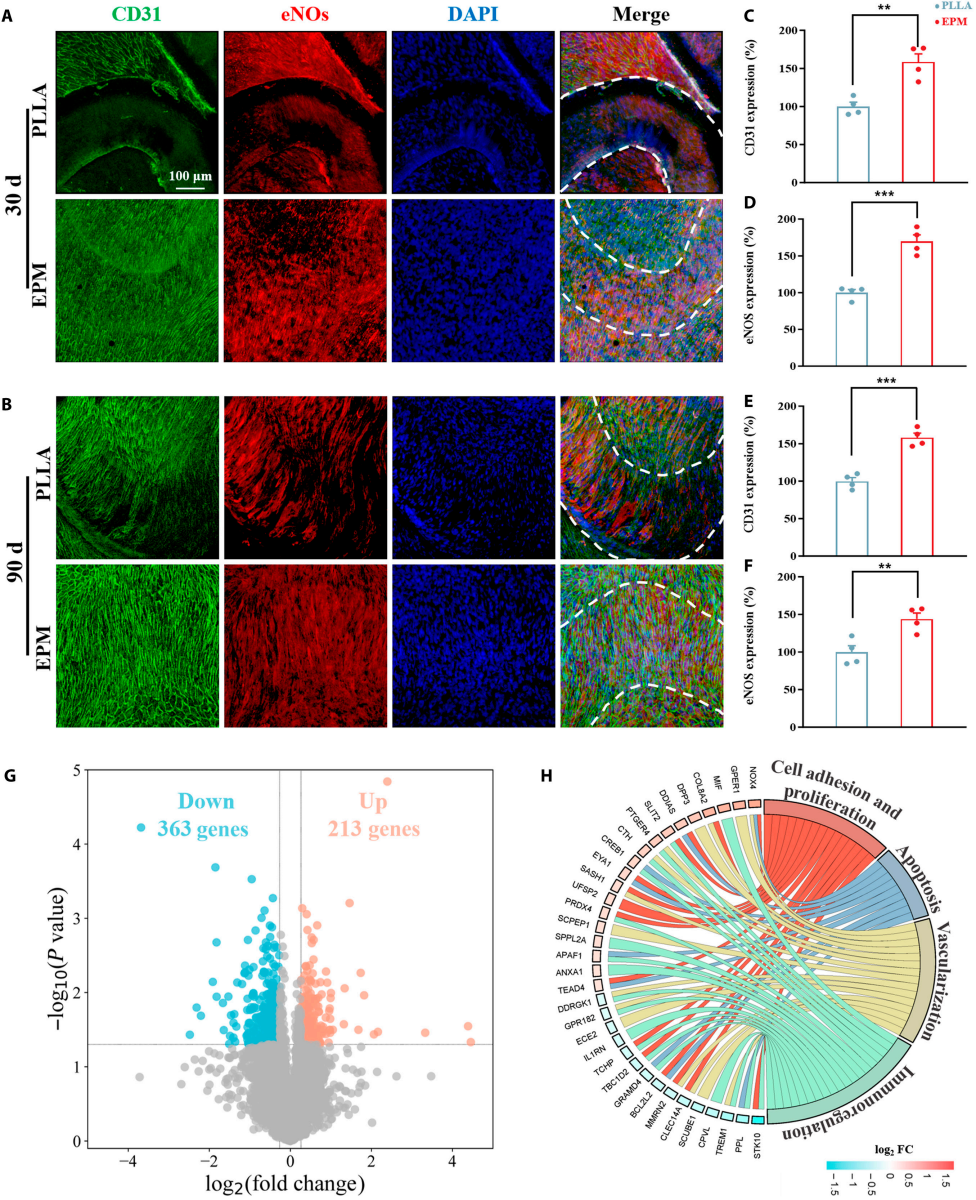
Vascular stent deployment in rabbits. Immunofluorescence analysis of the inner surface of the gathered vascular tissue following (A) 30 and (B) 90 d of implantation. The struts of the stent are outlined in white dashed lines. Quantitative analysis of CD31 intensity on the neointimal surface following (C) 30 and (E) 90 d of implantation. Quantitative analysis of endothelial nitric oxide synthase (eNOS) intensity following (D) 30 and (F) 90 d of implantation (*n* = 4). (G) Volcano plot showing differentially expressed genes of the stented aortas. Genes meeting the differential expression criteria (|log_2_ FC| > 0.263 and *P* < 0.05) are marked in blue for down-regulation and orange for upregulation. (H) Circular plot displaying the results of the gene-annotation enrichment analysis (*n* = 4). FC, fold change.

Immunofluorescence staining was employed to assess the ECs on the neointimal surface of the implanted stents. Marker proteins CD31 and endothelial nitric oxide synthase (eNOS), which are expressed by vascular ECs, are integral for maintaining endothelial function and vascular homeostasis [67]. Immunofluorescence staining for CD31, eNOS, and nuclei on the neointimal surface was carried out (Fig. 8A and B), and CD31/eNOS signals were quantified (Fig. 8C to F), with that of the bare PLLA stent group set at 100%. The incomplete endothelialization observed in the bare PLLA group at 30 d indicated a delay in endothelial recovery. In contrast, at both 30 and 90 d, stents coated with EPM exhibited significantly enhanced CD31 and eNOS expression and complete endothelial coverage compared to the bare PLLA stents, thereby promoting rapid restoration of the endothelial layer. This improved neointimal function may result from the synergistic action of multiple platelet proteins.

Collectively, these findings suggest that EPM-coated stents not only mitigate early inflammatory responses but also support long-term vessel patency and normal endothelial development in vivo, underscoring their potential to facilitate optimal vascular remodeling.

RNA sequencing analysis of gene expression in vascular tissues surrounding stents at 21 d postimplantation revealed a markedly distinct profile in the EPM-treated group compared to that in bare stents. The volcano plot (Fig. 8G) shows that 213 genes were upregulated and 363 genes were down-regulated beyond the threshold of |log_2_ FC| > 0.263 and *P* < 0.05. Functional enrichment via the UniProt database classified these genes into key biological processes, including cell adhesion and proliferation, apoptosis, vascularization, and immunoregulation (Fig. 8H). Detailed gene nomenclature is provided in Table S2, offering a comprehensive view of the genetic landscape modified by EPM treatment.

Notably, the upregulation of the BCL2L2 gene—which regulates apoptosis by inhibiting SMC death—may delay neovascular endothelial remodeling [68]. Similarly, increased expression of PTGER4, a gene involved in prostaglandin signaling, indicates enhanced anti-inflammatory responses in the EPM group [69]. In contrast, the down-regulation of genes such as NOX4, which mediates oxidative stress responses, suggests a reduction in oxidative damage, creating a more favorable environment for vascular repair [70]. Changes in the expression of genes like SASH1 (related to cell adhesion) [71] and IL1RN (an immune response modulator) [72], as well as alterations in PRKCB—an important mediator of cellular signaling [73]—reflect dynamic shifts in cell interactions and inflammation within the stented areas. Furthermore, the upregulation of TEAD4 [74] and COL8A2 [75] suggests a propensity to promote cell proliferation and extracellular matrix formation, processes that are conducive to vascular repair.

In essence, these findings indicate that EPM-coated stents foster a tissue environment characterized by reduced inflammatory responses, diminished oxidative stress, and enhanced remodeling capacity. Such conditions are likely to maintain the integrity and functionality of the vascular system postimplantation, potentially leading to improved long-term outcomes and fewer complications associated with stent therapy.

## Conclusion

In conclusion, the development and characterization of EPM coatings for vascular stents have achieved encouraging results in creating biocompatible interfaces and enhancing cellular responses. The uniform EPM coatings, established through the functional groups present on the platelet membrane and stabilized by chemical cross-linking with EGCG, have demonstrated a strong capability to retain biofunctional proteins, thereby preserving the inherent antithrombotic and anti-inflammatory properties of the platelet membrane.

The successful integration of EPM coatings on diverse substrates highlights their broad applicability. Both in vitro and ex vivo hemocompatibility tests reveal marked reductions in platelet adhesion and activation, indicating an enhanced anticoagulant profile that is critical for minimizing thrombus formation post-stenting. Additionally, cytocompatibility assessments confirm that these coatings support EC proliferation while curbing excessive SMC growth, potentially reducing the risk of ISR. The coatings also induce a favorable inflammatory milieu by diminishing macrophage adhesion and promoting an anti-inflammatory phenotype. In vivo results further substantiate the coatings’ ability to maintain long-term stent patency and to accelerate rapid endothelialization—essential factors in averting late-stent thrombosis and restenosis. Moreover, the diverse gene expression profiles associated with EPM-coated stents underscore their capacity to orchestrate complex biological interactions that beneficially modulate inflammation, cellular remodeling, and oxidative stress responses.

In considering the macroscale application of platelet membrane coatings, the use of autologous platelet sources is a critical aspect due to its potential to mitigate immune rejection, thus enhancing the biocompatibility and bioactivity of the implants [76,77]. This approach not only ensures better integration with the host but also alleviates ethical concerns regarding donor blood sources. However, the bulk extraction of platelet membranes poses challenges related to the efficiency and economic feasibility of the separation and purification processes, particularly at commercial scales that demand high purity and quality consistency [78]. Furthermore, the collection and processing of autologous platelets require specific medical procedures, which may limit widespread application. Individual variability in platelet quality and quantity can also affect the consistency of the research and its applications. Addressing these challenges is crucial, and future research should focus on developing more efficient and cost-effective methods for the preparation and use of autologous platelet membranes to pave the way for clinical translation.

Nevertheless, the multifunctional biointerface achieved through EPM coatings represents a notable advancement in stent design, with the potential to improve the healing process and long-term efficacy of stent therapy. Such protected armor for vascular stents successfully presented a balance among anticoagulant, anti-inflammatory, and pro-endothelial functions. The establishment of stable cell membrane coatings also opens up expansive application possibilities in various fields, providing an important benchmark for subsequent modifications of medical devices.

## Materials and Methods

### Materials and ethical issues on animal experiments

The protease inhibitor cocktail was obtained from APExBIO (USA). Dopamine hydrochloride and EGCG were obtained from Aladdin Bio-Chem Technology Co. Ltd. Heparin sodium salt (150 U/mg) was purchased from Macklin Biochemical Co. Ltd. Pentobarbital sodium salt was purchased from TCI. The PLLA sheets and stents were provided by Sichuan Xingtai Pule Medical Technology Co. Ltd. The PVC sheets were purchased from Tuyang Health Products Store. PET and PC sheets were purchased from Shenzhen Yingjiada Insulating Plastic Materials Co. Ltd. Si wafers were purchased from Ningbo Yilin Semiconductor Technology Ltd. Ti foils and 316L stainless steel foils sheets were purchased from Hefei Wenghe Metal Materials Co. Ltd. Unless stated otherwise, all other reagents were analytical grade and procured from Kelong Regents Company. Hematocompatible and cytocompatible biological reagents were purchased from specialized suppliers, and their details are provided in the relevant sections describing the subsequent experiments. All animal experiments were approved by the Animal Ethics Committee of West China Hospital, Sichuan University (Sichuan Medical Ethics Committee, K2023010).

### Preparation of EGCG and EPM coatings

PMVs were prepared with reference to previous literature [42]. Briefly, platelet-rich plasma from whole blood was obtained by centrifugation, further centrifuged to collect platelets, and frozen after adding the protease inhibitor cocktail. Platelet membranes were first disrupted by multiple freeze–thaw cycles and then washed several times with PBS to remove soluble cytoplasmic components. Taking PLLA as an example, the substrate was ultrasonically cleaned 3 times with ethanol and deionized water, respectively, before coating. Firstly, the substrate was immersed in 2 mg/ml dopamine Tris solution (pH = 8.5, 50 mM) for 2 h. After the end of the reaction, the substrate was ultrasonically cleaned for 3 min in a water-bath ultrasonic pot and then cleaned with ultrapure (UP) water 3 times, and air-dried to obtain PDA coating.

A solution of 1 mg/ml EGCG Tris (pH = 8.5, 50 mM) was mixed with UP water in equal proportions and then added to the surface of the PDA coating to form a uniform liquid film on the surface of the coating. A solution of 1 mg/ml EGCG Tris (pH = 8.5, 50 mM) was mixed with a suspension of PMVs with a protein content of 1 mg/ml in equal proportions and then added to the surface of the PDA coating. After incubation at 37 °C for 2 h, the surface was then washed with UP water to obtain EGCG and EPM coatings. If not specified, EPM coatings prepared for subsequent experiments were derived from rabbit platelets.

In order to verify their multifunctionality, EPM coatings were also prepared on other substrates (including PC, PET, PVC, Ti foil, 316L stainless steel foil, and Si sheets) through the same method as that for PLLA substrates.

The PLLA stents were cleaned, and the PDA coating was prepared as previously described. The stents were then submerged in a mixture of 2 solutions: a suspension of PMVs and a solution of 1 mg/ml EGCG Tris (pH = 8.5, 50 mM) in equal proportions. After a 30-s incubation period, the stents were lifted and dried at 37 °C for 15 min. The process was repeated 5 times. The mixed solution of EGCG and cell membrane vesicles was prepared freshly each time.

### Physiochemical characterization of EPM coatings

The surface morphology of the coatings was observed by SEM (Phenom ProX, Thermo Scientific). The surface elemental composition was further investigated by x-ray photoelectron spectroscopy (XSAM800, Kratos) using an Al Kα x-ray source (1,486.6 eV). Rhodamine 6G-labeled coatings were observed by CLSM at an excitation wavelength of 556 nm (LSM 880, Axio Observer, Zeiss). Silicon wafers with surface-prepared EPM were cleaved from the backside to examine the cross-section. The thickness of the coating was measured using SEM, with the interface between the silicon substrate and the coating serving as the reference point. After cleavage, the fractured silicon wafer was stained with rhodamine, and fluorescence images were collected. The thickness of both the dry and wet states of EPM coatings were determined using an ellipsometer (UVISEL Plus, Horiba Scientific). For the wet state, before measurement, PBS solution was applied to the surface of the sheet for 3 min. The mechanical properties and surface morphology of the samples were characterized using an atomic force microscope (Dimension ICON, Bruker) operated in PeakForce Tapping mode. The surface Young’s moduli of the samples in the wet states were measured using an SNL-10 probe, while measurements of surface morphology under dry conditions were performed with an RTESPA-300 probe. The hydrophilicity change of the coatings was assessed by measuring the static water contact angle (Theta Lite, Biolin Scientific).

#### SDS-PAGE and Western blotting analysis

The PMVs used in this experiment were derived from rat platelets. PMV and EPM powders were suspended in SDS-PAGE loading buffer (Beyotime Biotechnology). The mixtures were then applied to a 10% Bis-Tris gel, and proteins were separated in running buffer (Solarbio Science & Technology) using a Bio-Rad electrophoresis system. Electrophoresis proceeded with the same SDS-PAGE loading buffer. After transferring proteins to polyvinylidene fluoride membranes (Bio-Rad Laboratories), the membranes were blocked in Tris-buffered saline with Tween-20 using 5% skim milk or bovine serum albumin and incubated with appropriate primary and secondary antibodies. Protein bands were visualized using an enhanced chemiluminescence kit and the ChemiDoc XRS+ system (Bio-Rad Laboratories). The primary antibodies used in this study included CD47 rabbit polyclonal antibody (pAb; ABclonal), CD55 rabbit pAb (ABclonal), CD59 rabbit pAb (ABclonal), CD62P rabbit monoclonal antibody (mAb; ABclonal), integrin α2 rabbit mAb (ABclonal), integrin β1/CD29 rabbit mAb (ABclonal), and goat anti-rabbit immunoglobulin G (H+L) (Affinity).

#### Coating stability testing

Ti samples with surface-prepared EPM were loaded into PVC tubing and flushed through a peristaltic pump at a flow rate of 70 cm/s in a flowing PBS solution environment, thus simulating a blood flow environment. Ti samples were used in stability testing and the following ex vivo circulation experiments to facilitate inserting surface-treated Ti foils into the circulation tubing. After 8, 16, 24, and 32 d, the samples were removed, stained with rhodamine 6G, and visualized with CLSM. The fluorescence intensity of freshly prepared EPM coatings was set at 100% by calculating the fluorescence intensity of residual EPM to quantify the coating residue. Silicon wafers treated with EPM on their surfaces were loaded into PVC tubing after being cleaved and flushed in a flowing PBS solution environment. After 4, 8, 12, and 16 d, the samples were removed and the thickness of the coatings was measured using SEM.

### Blood compatibility test

#### In vitro platelet adhesion test

Two weeks prior to conducting the experiments, fresh rabbit blood from healthy New Zealand rabbits was taken for EPM coating preparation. Blood from corresponding New Zealand Large White rabbits was taken and anticoagulated with sodium citrate. PLLA sheets (1 cm × 1 cm) were placed into 24-well plates and incubated with 500 μl of platelet-rich plasma at 37 °C for 1 h and then rinsed with PBS to remove nonadherent platelets. Afterward, they were fixed with glutaraldehyde solution (2.5%) overnight, and the in vitro hemocompatibility of the EPM coating was evaluated by SEM observation after gradient dehydration.

#### Ex vivo antithrombotic evaluation of the arteriovenous shunt method

Two weeks before conducting the experiments, corresponding adult New Zealand Large White rabbits (2.5 to 3 kg, ~3 months of age), provided by Chengdu Dorsey Laboratory Animal, were selected and fresh rabbit blood was extracted for the preparation of the EPM coating. Briefly, uncoated and EGCG- and EPM-coated Ti samples were rolled up and placed into different parts of the PVC catheter, which were then connected in parallel and assembled with an indwelling needle. The ear vein of the corresponding rabbit was anesthetized with sodium pentobarbital (25 mg/ml, 1.0 ml/kg). One side of the carotid artery and the other side of the external jugular vein were isolated from adult New Zealand White rabbits, and then the vessels were connected with a catheter to establish an extracorporeal circulation pathway. After 1 h, the catheter’s cross-section was photographed to quantify the extent of circuit occlusion. Samples were then removed, flushed with 0.9% saline, fixed, imaged again, dehydrated, and weighed.

### Cell culture

Human umbilical vein ECs (Cat. No. STCC12103) and umbilical artery SMCs (Cat. No. HTX2180) were obtained from Service-bio Technology Co. Ltd., while murine monocytes (RAW264.7; Cat. No. SCSP-5036) came from the Chinese Academy of Sciences Cell Bank. All lines were maintained in Dulbecco’s modified Eagle medium (DMEM) containing 10% (v/v) fetal bovine serum (Gibco) and 1% (v/v) penicillin–streptomycin (Gibco), at 37 °C in a 5% CO_2_ incubator until ~80% confluence.

For subculturing or in vitro vascular cytocompatibility assays, ECs and SMCs were rinsed with PBS and incubated with 0.05% (w/v) trypsin/0.53 mM EDTA (Gibco) to detach them; then, the cell suspension was collected and centrifuged, and the pellet was resuspended in fresh medium. RAW264.7 cells were processed similarly: the old medium was discarded, cells were harvested by centrifugation, and the pellet was resuspended in new DMEM for further passaging or cytocompatibility testing.

Uncoated and EGCG- and EPM-coated PLLA slices (1 cm × 1 cm) were loaded into 24-well culture plates (NEST Biotechnology) and sterilized by ethylene oxide at 37 °C for 12 h; 1-ml suspensions of ECs or SMCs containing 2 × 10^4^ cells were inoculated onto the surface of the samples and cultured for 24 and 72 h. Cell proliferation was assessed by CCK-8 assay to assess cell proliferation, and adherent cells on surfaces stained with fluorescein diacetate (Merck) were visualized by fluorescence microscopy (DMI4000B, Leica). A 30 μg/ml fluorescein diacetate solution (500 μl in PBS) was added to each well of a 24-well plate and incubated for 5 min for staining. Cell proliferation on the substrates was then quantified by a CCK-8 metabolic assay (fresh basal medium mixed with CCK-8 reagent at a 9:1 v/v ratio), and absorbance was measured at 450 nm.

Uncoated and EGCG- and EPM-coated PLLA slices (1 cm × 1 cm) were loaded into 24-well culture plates and sterilized by ethylene oxide at 37 °C for 12 h, with the EPM coating prepared using rat PMVs as the source material; 1 ml of RAW264.7 suspension containing 4 × 10^4^ cells was inoculated onto the surface of the samples, and the samples were incubated for 24 h. Tetramethylrhodamine isothiocyanate-conjugated phalloidin (TRITC phalloidin; Solarbio) and 4′,6-diamidino-2-phenylindole (DAPI; Solarbio) were used to visualize F-actin and nuclei, respectively. Cells were fixed in 4% paraformaldehyde for 15 min, permeabilized with 0.5% Triton X-100 for 5 min, and then incubated with TRITC phalloidin (5 μg/ml in PBS) for 30 min and DAPI (10 μg/ml in PBS) for 1 min. Adherent cells on the samples were visualized by CLSM. Meanwhile, the TNF-α and IL-10 concentrations in the culture supernatants were measured using ELISA kits (Jingmei Biotechnology).

EGCG and EPM coatings, which were prepared from rat platelet vesicles, were prepared separately in 6-well cell culture plates (NEST Biotechnology) and sterilized by ethylene oxide at 37 °C for 12 h. The wells with no treatment were used as controls. To assess macrophage polarization, 5 ml of RAW264.7 suspension containing 2 × 10^4^ cells was inoculated into 6-well plates after 36 h of incubation, and the cell receptors were treated with APC Anti-Mouse CD86 Antibody (Elabscience Biotechnology) and PE Anti-Mouse CD206 Antibody (Elabscience Biotechnology), followed by a cell flow assay to analyze the polarization of M1- and M2-type macrophages.

### In vivo subcutaneous implantation

Male SD rats (about 10 weeks old, 250 to 300 g), obtained from Chengdu Dorsey Laboratory Animal Ltd., were used for subcutaneous implantation. Under anesthesia (intraperitoneal sodium pentobarbital, 20 mg/ml at 0.25 ml/100 g), the dorsal skin was carefully separated from the underlying muscle. Uncoated and EGCG/EPM-coated PLLA slices were then implanted on either side of the dorsum (3 implants per animal). At 14 and 28 d postimplantation, the fibrous capsules surrounding each sample were collected, and the rats were euthanized by pentobarbital overdose. Excised tissues were fixed and stained with H&E, and immunofluorescence (TNF-α [Abcam] and IL-10 [Abcam]).

### In vivo stent implantation

For the in vivo vascular stent implantation experiments, male New Zealand Large White rabbits (about 3 months old, 2.5 to 3 kg) were employed. Two weeks prior to the experiment, the same volume of blood was collected from all rabbits to undergo stent implantation, some of which were subsequently targeted for implantation of EPM stents prepared from their own platelets. The extracted platelet membranes were then used to prepare the coating. The study involved implanting an uncoated stent into the celiac artery of rabbits, while the EPM stent was implanted into the corresponding rabbits. The control group received a PLLA stent. General anesthesia in rabbits was achieved by infusing sodium pentobarbital (25 mg/ml, 1.0 ml/kg) through the ear vein. A 3-cm segment of the femoral artery was then exposed and prepared for stent deployment. Using a balloon catheter, the stent was advanced into the abdominal aorta and expanded-balloon inflation was held at 8 atm for 40 s to secure the device in place.

After 6 h, a batch of stents implanted into the vessels was removed to study acute thrombosis of the stents. It is important to note that this batch of rabbits did not receive any antithrombotic drugs. The harvested vascular stents were fixed, dehydrated, and observed for stent morphology using SEM.

After stent implantation, the rabbits received intramuscular injection of penicillin sodium solution (300,000 U/ml, 0.2 ml/kg) and oral administration of aspirin (2.5 mg/kg) and clopidogrel (3 mg/kg) for 3 consecutive days. The stented aortic tissue was removed 21 d after implantation, and the rabbit was euthanized with a pentobarbital sodium salt solution overdose. The vascular stent samples were frozen in liquid nitrogen. Concentrations were measured using NanoDrop ND-1000. From each sample, 1 to 2 μg of total RNA was selected to construct RNA sequencing libraries. The quality of the libraries was assessed using Agilent 2100 Bioanalyzer, and quantification was performed by the quantitative real-time polymerase chain reaction. An Illumina NovaSeq 6000 sequencer was used for sequencing and analysis. Library construction and sequencing programs were provided by Aksomics Inc.

After 30 and 90 d of implantation, the aortic tissues with stents were removed and fixed with a 4% formaldehyde solution. The excised aortic tissue was split into 3 portions for immunofluorescence staining, immunohistochemical analysis, and SEM examination. Vascular stent tissues were stained with H&E, CD68 antibody (Abcam), α-SMA antibody (Abcam), CD86 antibody (Biosynthesis Biotechnology), CD206 antibody (Cell Signaling Technology), CD31 antibody (Abcam), and eNOS antibody (Abcam). Subsequently, we performed H&E staining to calculate the neointimal stenosis ratio. We further quantified the expression levels of CD68, α-SMA, CD86, CD206, CD31, and eNOS in the neointima. Protein expression was quantified as the mean fluorescence intensity, determined by measuring the tissue’s fluorescence signal and normalizing it to a unit area.

### Statistical analysis

All experiments in this paper were executed with a minimum of 3 independent replicates, with quantitative outcomes expressed as mean value ± standard deviation. Statistical analysis was performed using GraphPad Prism 9.0. For comparative analyses between 2 experimental groups, independent 2-tailed Student *t* tests were implemented. Additionally, one-way analysis of variance was employed to discern differences across multiple groups. Statistical significance was demarcated as follows: *P < 0.05, **P < 0.01, ***P < 0.001, and ****P < 0.0001, while “ns” denotes nonsignificant differences (*P* ≥ 0.05).

## Data Availability

The data are freely available upon request.

## Supplementary Materials

Supplementary 1 Description of Supplemental Experiment Figs. S1 to S8 Tables S1 and S2
