# Supplementary material for: A Versatile Macroscale Platelet Membrane Coating: A Biomimetic Strategy to Balance Antithrombogenicity and Proendothelialization on Vascular Stents
Source: Research (Wash D C). 2025 Dec 12;8:1007. doi: 10.34133/research.1007 (PMC12698931; doi:10.34133/research.1007)
Supplement: Supplementary 1 — Description of Supplemental Experiment Figs. S1 to S8 Tables S1 and S2 [file research.1007.f1.zip › supplemental information.docx]

**Supplemental Information**

**A Versatile Macroscale Platelet Membrane Coating: A Biomimetic Strategy to Balance Antithrombogenicity and Proendothelialization on Vascular Stents**

Yongqi An^1^, Cheng Ji^1^, Hao Zhang^3^, Hui Yan^1^, Dimeng Wu^1,4^, Rifang Luo^1,*^, Yunbing Wang^1,2,4^

1. National Engineering Research Center for Biomaterials, Sichuan University, Chengdu 610064, China

2. Research Unit of Minimally lnvasive Treatment of Structural Heart Disease, Chinese Academy of Medical Sciences (No: 2021RU013), Beijing 100730, China

3. Vanadium and Titanium Critical Strategic Materials Key Laboratory of Sichuan Province, Panzhihua University, Panzhihua 617000, China

4. Chengdu Minshan Institute of Biomaterials, Chengdu 610213, China

*: Corresponding Author E-mail address: lrifang@scu.edu.cn

**Contents: Figure S1 to S8, Table S1 and S2**

**Description of Supplemental Experiment:**

**Preparation and characterization of PMVs.**

Briefly, platelet-rich plasma from whole blood was obtained by centrifugation, further centrifuged to collect platelets, and frozen after adding PI. Platelet membranes were first disrupted by multiple freeze-thaw cycles and then washed several times with PBS to remove soluble cytoplasmic components. The membrane fragments were finally suspended in water and sonicated to obtain PMVs. The morphology of the vesicles was observed by transmission electron microscopy (TEM, JEM-2100 PLUS, Nihon Electronics JEOL). The vesicle size and zeta potential were measured by dynamic light scattering (DLS, Zetasizer Nano-ZS90, Malvern).

**Determine the optimal concentration of E_m_PM_n_ coatings.**

Firstly, the PLLA substrate was immersed in 2 mg/mL dopamine Tris solution (pH=8.5, 50 mM) for 2 h. After the end of the reaction, the substrate was ultrasonically cleaned for 3 min in a water-bath ultrasonic pot, and then cleaned with UP water for three times, and air-dried to obtain the PDA coating. 1 mg/mL EGCG Tris solution (pH=8.5, 50 mM) was mixed with UP water in equal proportions, and then added to the surface of the PDA coating to form a uniform liquid film on the surface of the coating. Tris solution of 2m mg/mL EGCG (pH=8.5, 50 mM) was mixed with a suspension of PMVs with a protein content of 2n mg/mL in equal proportions, and then added to the surface of the PDA coating. Incubate at 37 ℃ for 2 h, then wash with UP water to obtain EGCG and E_m_PM_n_ coatings.

Nano-scratch testing of E_0.1_PM_0.5_ and E_0.5_PM_0.5_ coatings was carried out with the aim of evaluating whether the increase in the amount of EGCG affects the coating bond strength and scratch resistance. The scratch instrument (Hysitron TI 980 Triboindenter, Bruker) was set with a linear loading mode, a maximum load of 10 mN, a scratch length of 300 μm, and a scratching speed of 7.5 μm/s.

*In vitro* platelet adhesion test. Two weeks prior to conducting the experiments, fresh rabbit blood from healthy New Zealand rabbits was taken for EPM coating preparation. Blood from corresponding New Zealand Large White rabbits was taken and anticoagulated with sodium citrate. PLLA sheets (1 cm × 1 cm) were placed in 24-well plates and incubated with 500 µL of platelet-rich plasma at 37 ℃ for 1 h, then rinsed with PBS to remove non-adherent platelets. Afterwards, glutaraldehyde solution (2.5%) was fixed overnight, and the platelet adhesion of the E_m_PM_0.5_ coating was evaluated by SEM observation after gradient dehydration.

The stability of the different E_0.5_PM_n_ was monitored using a QSense Analyzer instrument (Biolin Scientific) according to the following protocol: (1) PDA was pre-deposited on a gold-plated quartz crystal (QSX 301, Biolin Scientific); (2) Tris solution was injected into the chamber at a rate of 30 μL/min for about 10 minutes; (3) the mixture of Tris solution of EGCG and a suspension of PMVs was injected into the chamber at 30 μL/min for about 30 minutes; (4) Tris solution was injected into the chamber at a rate of 30 μL/min for about 10 minutes. The concentrations of the aforementioned solutions were identical to those employed in the experimental procedure, and all measurements were conducted at room temperature.

**Results and discussion**

We first isolated PMVs and observed the PMVs from rabbits by TEM (**Fig. S1a**). The hydrated particle size and zeta potential (**Fig. S1b**) of the prepared rabbit platelet vesicles were measured to be 225.9 ± 2.3 nm and -21.2 ± 0.2 mV, respectively. Similarly, the rat platelet vesicles exhibited a hydrated particle size of 232.1 ± 1.8 nm and a zeta potential of -21.8 ± 0.2 mV. The closely matched sizes and surface potentials indicate that the physicochemical properties of the two types of PMVs are highly consistent.

When the platelet membrane coating was prepared, SEM results (**Fig. S2a**) showed that the platelet membrane coating without EGCG was loose, with weak inter-coating cohesion, and the loose structure was easy to be damaged when squeezed or washed by mechanical force; whereas the cell membrane coating with EGCG cross-linking (**Fig. S2b**) showed a homogeneous surface, and the scratch test also showed that the inter-coating cohesion was stronger, and the stability of the coating was enhanced ^1^.

An attempt was made to fabricate multilayer platelet membrane coatings on the same substrate. The results (**Fig. S3**) demonstrated that the second cell membrane layer maintained excellent integrity, indicating that the process allows for controlled modulation of the cell membrane coating.

In this study, EGCG was employed both as an anchoring agent for the substrate and as a facilitator in the coupling of PMVs. The amount of EGCG must be precisely controlled: insufficient levels may fail to enhance the uniformity and stability of the cell membrane coating, while excessive amounts can impair cell membrane fusion and alter the coating’s composition. Experiments were performed on various E_m_PM_n_ coatings, where m denotes the final concentration of EGCG and n represents the final concentration of PMV protein. The concentration of platelet membrane vesicles was fixed at 0.5 mg/mL, and coatings with varying EGCG concentrations — namely, E_0.1_PM_0.5_, E_0.5_PM_0.5_, E_1.0_PM_0.5_, and E_5.0_PM_0.5_ — were fabricated on PLLA substrates. SEM images (**Fig. S4a**) revealed that E_0.1_PM_0.5_, E_0.5_PM_0.5_, and E_1.0_PM_0.5_ formed a fully covered coating, but some nano-aggregate were observed on the surface of E_0.1_PM_0.5_, which were presumed to be dopamine particles. Additionally, it was determined the surface of E_1.0_PM_0.5_ exhibited greater roughness than that of the E_0.5_PM_0.5_. This observation may be attributed to localized excessive oxidative polymerization, a phenomenon potentially induced by EGCG. Numerous pores were detected on the surface of E_5.0_PM_0.5_, likely attributable to robust oxidative polymerization induced by a high concentration of EGCG. This process resulted in excessive cross-linking and enhanced interactions at the cell membrane interface, which prevents fusion of the cell membrane. This in turn affected the formation of subsequent homogeneous and dense macroscale cell membrane coatings ^2^. Nano-scratch testing of the coatings further corroborated that adjusting the EGCG loading modulates both the coating’s cohesive integrity and its adhesion to the underlying substrate. Specifically, increasing the EGCG concentration from 0.1 to 0.5 mg/mL led to a significant rise in the normal force required to initiate coating failure (**Fig. S4b**), demonstrating that higher EGCG content markedly enhances overall coating stability. Subsequently, a further evaluation of the platelet adhesion was conducted. SEM images (**Fig. S4c**) demonstrated, compared with the PLLA and EGCG samples, the E_0.1_PM_0.5,_ E_0.5_PM_0.5_ and E_1.0_PM_0.5_ exhibited significantly reduced platelet adhesion, indicating that the cell membrane coating can effectively achieve anticoagulation. The percentage composition of C, O, N, and P elements of the different coatings was analyzed by XPS (**Table S1**). In comparison to EGCG, the percentage of P in the E_m_PM_0.5_ coating increased to approximately 1%, while the lowest percentage of C and the highest percentage of N were observed in E_0.1_PM_0.5_ and E_0.5_PM_0.5_, respectively. According to the XPS analysis, the N/P ratio decreases with increasing EGCG concentration. In the E_m_PM_0.5_ coatings, the high N/P ratio may correspond with good platelet adhesion. The superior integrity and anticoagulant function observed in E_0.5_PM_0.5_ and E_1.0_PM_0.5_ can be understood as a result of effective cross-linking between EGCG and membrane proteins, which improves the stability of the coating. However, while the N/P ratio in the E_5.0_PM_0.5_ sample is lower, the abundant platelet activation indicates that excessive EGCG may mask the surface characteristics of the membrane and lead to a loss of membrane function ^3^. In summary, a moderate increase in EGCG concentration can enhance the membrane's performance, ensuring better cell adhesion and anticoagulant attributes. In this study, the optimal final concentration of EGCG in the EPM coating was 0.5 mg/mL.

Next, the final concentration of EGCG was fixed at 0.5 mg/mL in order to investigate the impact of varying final concentrations of platelet membrane vesicles on coating formation. E_0.5_PM_0.25_, E_0.5_PM_0.5_, E_0.5_PM_1.0_, and E_0.5_PM_2.0_ coatings were fabricated on PLLA substrates under identical remaining conditions. SEM images (**Fig. S5a**) demonstrate the presence of a considerable number of dopamine particles on the surface of E_0.5_PM_0.25_, indicating incomplete coverage of the EPM coating due to low cell membrane usage. In contrast, the E_0.5_PM_0.5_, E_0.5_PM_1.0_, and E_0.5_PM_2.0_ coatings exhibited a uniform and complete coating build. To further substantiate these findings, QCM measurements were conducted (**Fig. S5b**). The system baseline was first established with Tris buffer, after which a mixture of 0.5 mg/mL EGCG and 0.25, 0.5, or 1.0 mg/mL platelet membrane vesicles was introduced. Once the resonance frequencies had stabilized, the Tris solution was passed again. The observed decrease in frequency indicated an increase in adsorbed mass on the chip surface ^4^, manifesting a clear positive correlation between platelet membrane vesicle concentration and the degree of adsorption. Furthermore, when the mixture of platelet membrane vesicles and EGCG was deposited onto a PDA-coated substrate, subsequent rinsing with Tris solution led to a slight mass loss in both the E_0.5_PM_0.25_ and E_0.5_PM_0.5_ groups, whereas the E_0.5_PM_1.0_ group exhibited a pronounced initial attachment. After a 10-minute rinse, the amount of adsorbed mass in the E_0.5_PM_1.0_ group converged to a level similar to that of E_0.5_PM_0.5_, indicating that an excessive concentration of platelet membrane vesicles does not facilitate stable adhesion. Consequently, a platelet membrane vesicle concentration of 0.5 mg/mL was deemed optimal, and the E_0.5_PM_0.5_ coating solution was selected as the EPM coating for subsequent experiments.

**
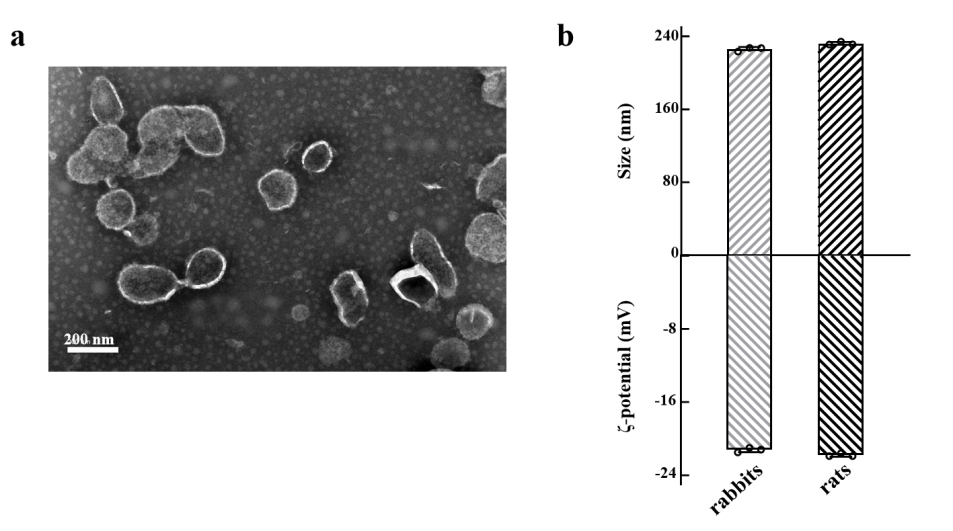
**

**Fig. S1. Preparation and characterization of PMVs. a)** TEM image of PMVs. **b)** Size and ζ-potential of PMVs from rabbits and rats.


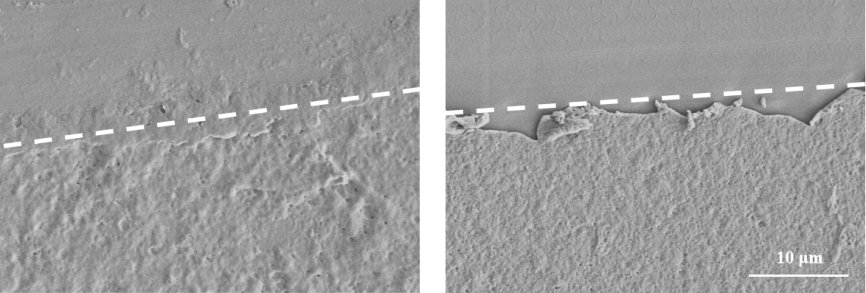


**Fig. S2. SEM images of platelet membrane coatings.** (**Left**) without EGCG and (**right**) with EGCG. The locations of the scratch are outlined by the white dotted line.


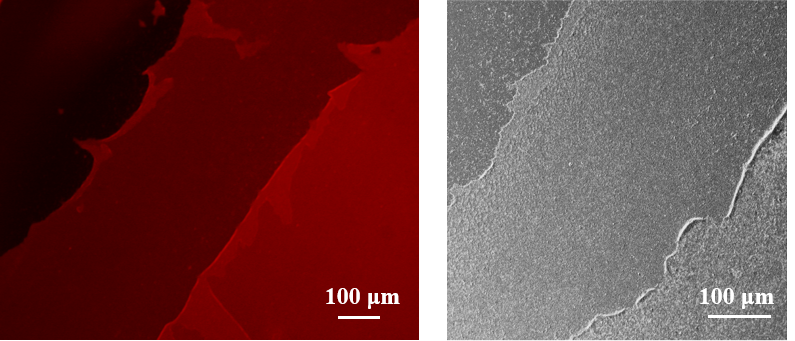


**Fig. S3. CLSM and SEM images of layered preparation of EPM coating.**


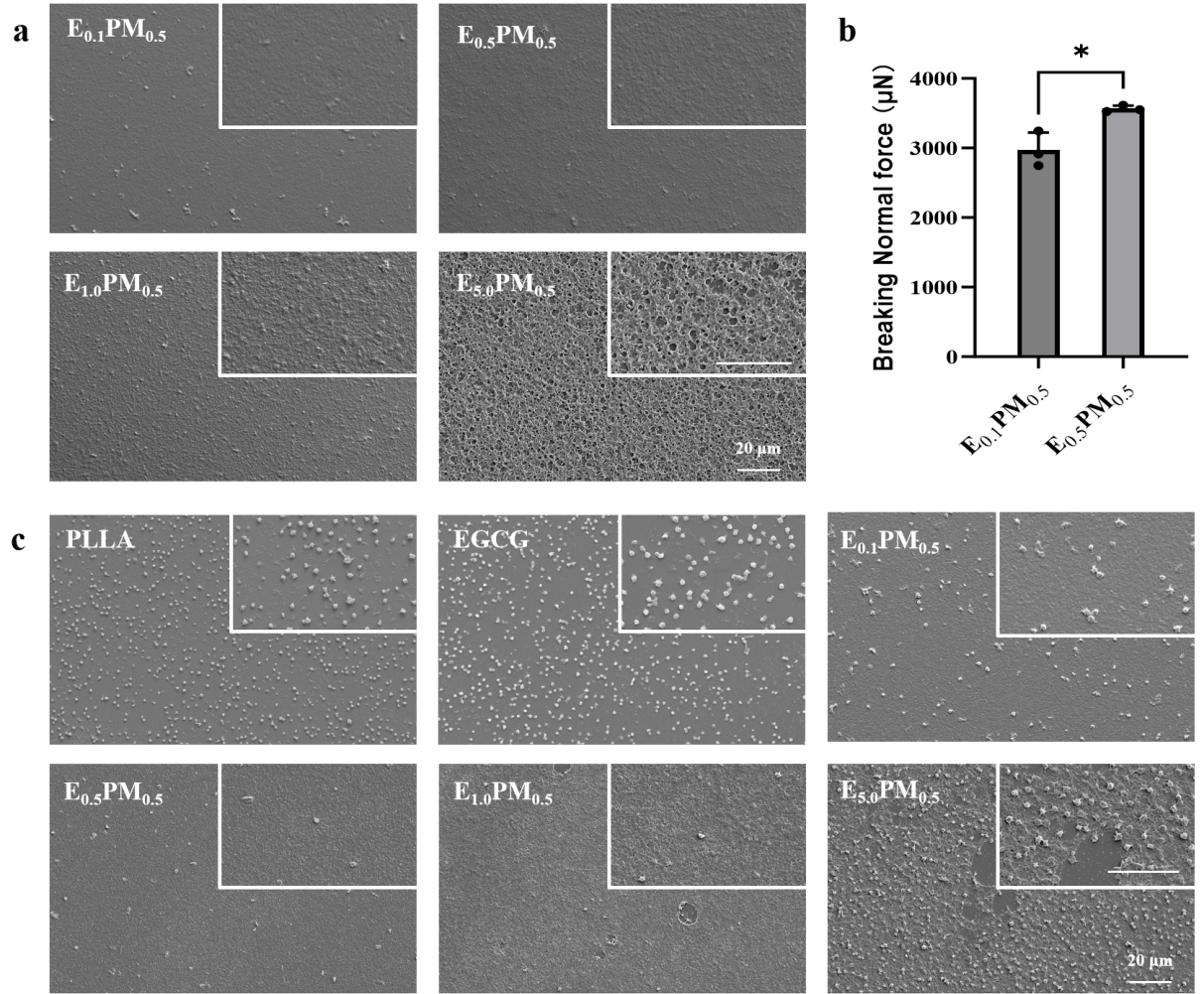


**Fig. S4.** Comparison between E_m_PM_0.5_ coatings. **a)** SEM images of EGCG and different E_m_PM_0.5_ coatings. **b)** Nano-scratch testing for E_0.1_PM_0.5_ and E_0.5_PM_0.5_ coatings (n=3). **c)** Morphology of adhered platelets on PLLA, EGCG and different E_m_PM_0.5_ coatings.

**Table S1. XPS spectra elemental ratio of EGCG and E_m_PM_0.5_ coatings.**

| **Element type** | **Elemental ratio(%)** | | | | |
| --- | --- | --- | --- | --- | --- |
|  | **EGCG** | **E_0.1_PM_0.5_** | **E_0.5_PM_0.5_** | **E_1.0_PM_0.5_** | **E_5.0_PM_0.5_** |
| C 1s | 77.26 | 75.78 | 75.79 | 76.21 | 76.27 |
| O 1s | 18.54 | 16.02 | 16.03 | 17.36 | 18.30 |
| N 1s | 4.10 | 7.38 | 7.32 | 5.54 | 4.62 |
| P 2p | / | 0.81 | 0.86 | 0.88 | 0.80 |


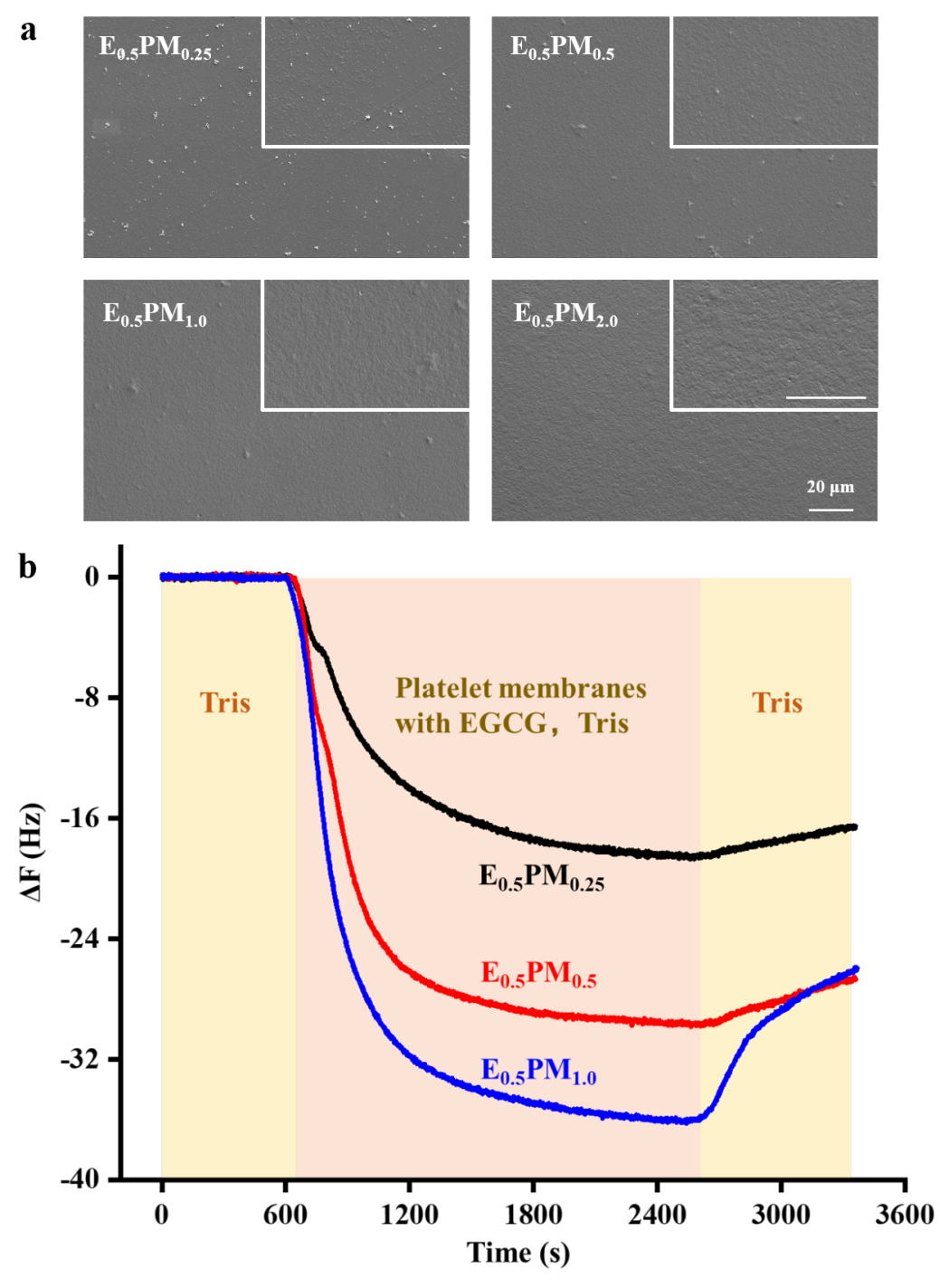


**Fig. S5.** **Comparison between E_0.5_PM_n_ coatings.** **a)** SEM images of different E_0.5_PM_n_ coatings. **b)** Real-time QCM monitoring of immobilization of different E_0.5_PM_n_ coatings on PDA-modified gold-plated quartz crystals.

**
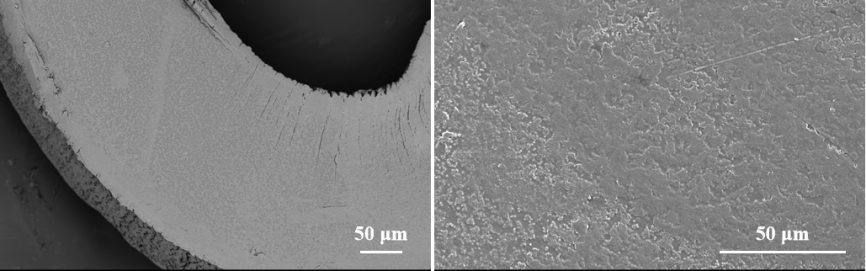
**

**Fig. S6.** SEM images of EPM-coated PLLA stents after balloon dilation in PBS at 37 °C.


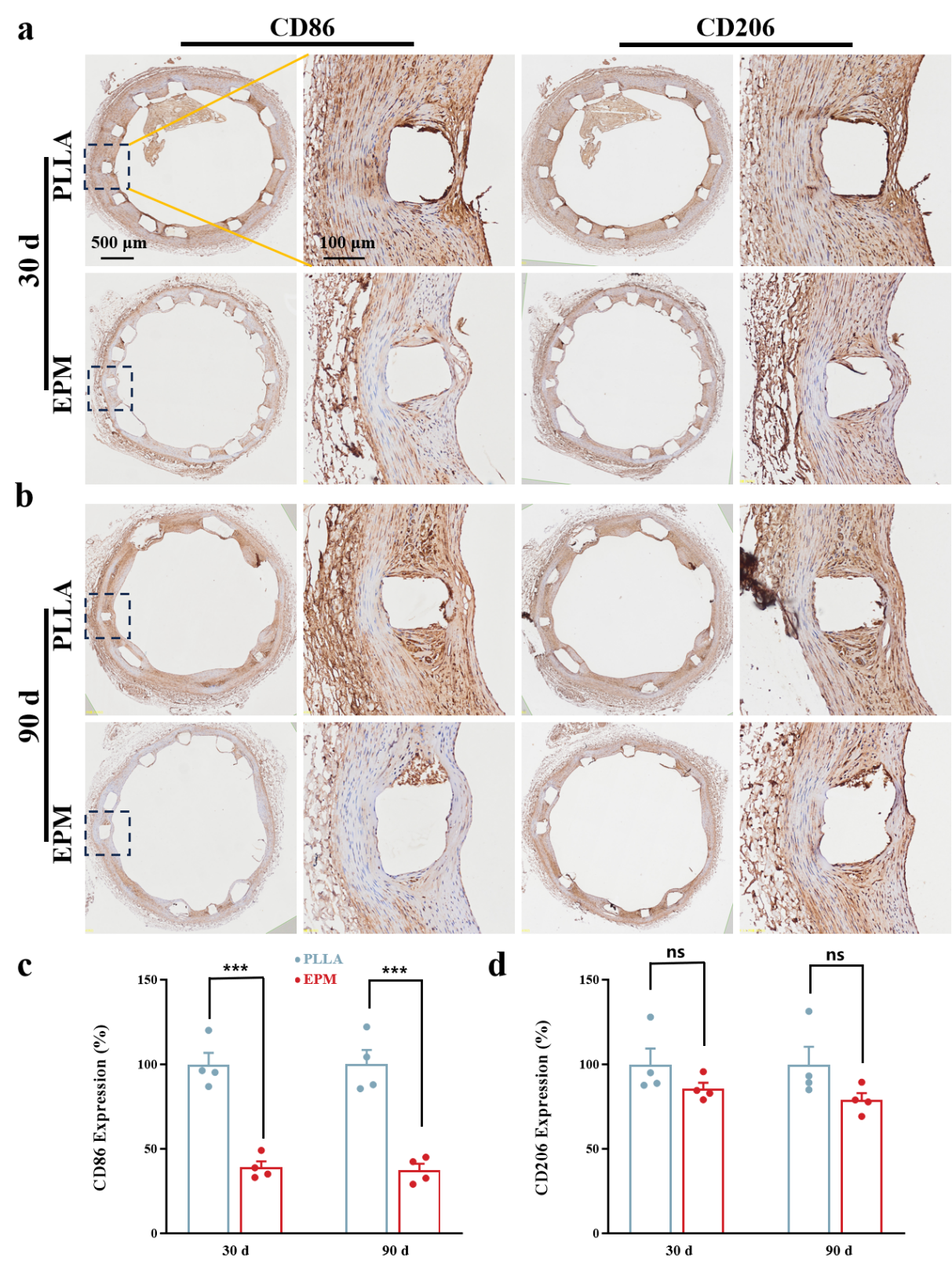


**Fig. S7. Vascular stent deployment in rabbits.** CD86 and CD206 staining of gathered vascular tissue following **a**) 30 and **b**) 90 days implantation. Quantitative analysis of **c**) CD86 expression, **d**) CD206 expression (n = 4).

**
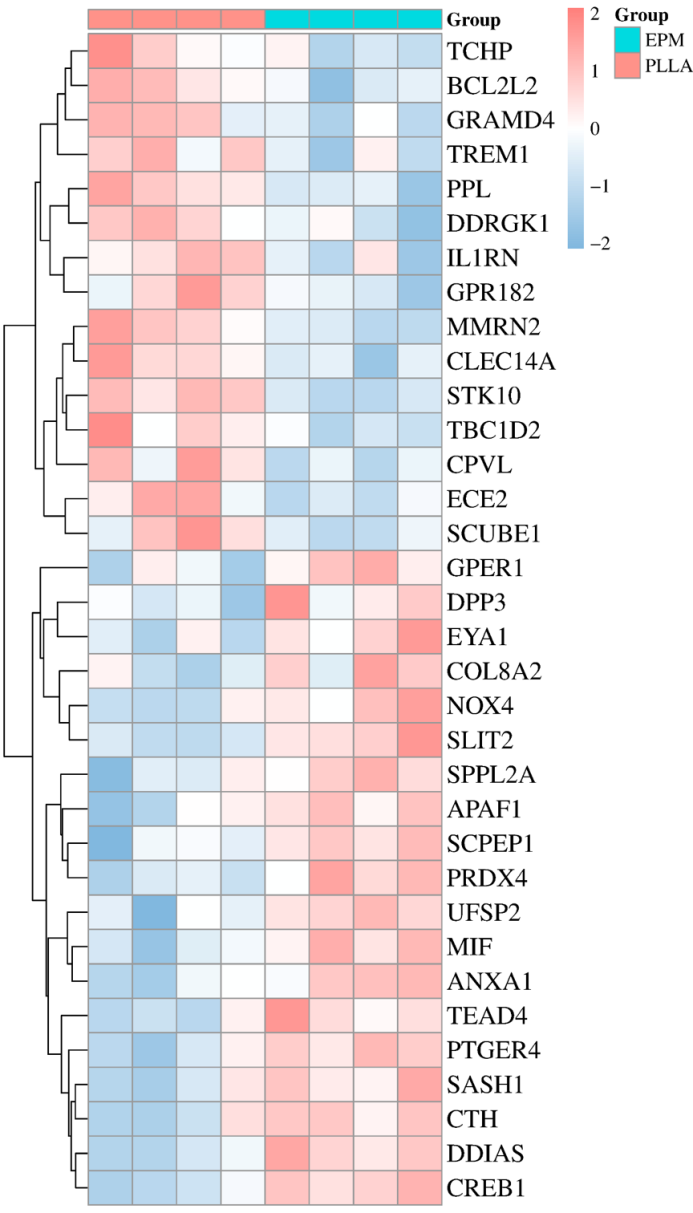
**

**Fig. S8. Heatmap of the partially differentially expressed genes between the PLLA and EPM groups.**

**Table S2. Abbreviations and full names of genes labeled in the stented aortas.**

| **Abbreviation** | **Full name** |
| --- | --- |
| ANXA1 | Annexin A1 |
| APAF1 | Apoptotic protease-activating factor 1 |
| BCL2L2 | Bcl-2-like protein 2 |
| CLEC14A | C-type lectin domain family 14 member A |
| COL8A2 | Collagen alpha-2(VIII) chain |
| CPVL | Carboxypeptidase, vitellogenic-like |
| CREB1 | Cyclic AMP-responsive element-binding |
| CTH | Cysteine-three-histidine |
| DDIAS | DNA damage-induced apoptosis suppressor |
| DDRGK1 | DDRGK Domain Containing 1 |
| DPP3 | Dipeptidyl peptidase 3 |
| ECE2 | Endothelin-converting enzyme 2 |
| EYA1 | Eyes absent homolog 1 |
| GPER1 | G-protein coupled estrogen receptor 1 |
| GPR182 | G-protein coupled receptor 182 |
| GRAMD4 | GRAM Domain Containing 4 |
| IL1RN | Interleukin-1 receptor antagonist |
| MIF | Macrophage migration inhibitory factor |
| MMRN2 | Multimerin 2 |
| NOX4 | NADPH oxidase 4 |
| PPL | Periplakin |
| PRDX4 | Peroxiredoxin-4 |
| PTGER4 | Prostaglandin E2 receptor EP4 subtype |
| SASH1 | SAM and SH3 domain-containing protein 1 |
| SCPEP1 | Serine carboxypeptidase 1 |
| SCUBE1 | Signal peptide, CUB and EGF-like domain-containing protein 1 |
| SLIT2 | Slit homolog 2 |
| SPPL2A | Signal peptide peptidase-like 2A |
| STK10 | Serine/threonine-protein kinase 10 |
| TBC1D2 | TBC1 Domain Family Member 2 |
| TCHP | Trichoplein keratin filament-binding protein |
| TEAD4 | TEA domain family member 4 |
| TREM1 | Triggering receptor expressed on myeloid cells 1 |
| UFSP2 | Ufm1-specific protease 2 |

**References**

(1) Xu, L. Q.; Neoh, K.-G.; Kang, E.-T. Natural polyphenols as versatile platforms for material engineering and surface functionalization. *Progress in Polymer Science* **2018**, *87*, 165-196. DOI: 10.1016/j.progpolymsci.2018.08.005.

(2) Chen, R.; Wang, J.-B.; Zhang, X.-Q.; Ren, J.; Zeng, C.-M. Green tea polyphenol epigallocatechin-3-gallate (EGCG) induced intermolecular cross-linking of membrane proteins. *Archives of Biochemistry and Biophysics* **2011**, *507* (2), 343-349. DOI: 10.1016/j.abb.2010.12.033.

(3) Sang, S.; Lambert, J. D.; Hong, J.; Tian, S.; Lee, M.-J.; Stark, R. E.; Ho, C.-T.; Yang, C. S. Synthesis and Structure Identification of Thiol Conjugates of (−)-Epigallocatechin Gallate and Their Urinary Levels in Mice. *Chemical Research in Toxicology* **2005**, *18* (11), 1762-1769. DOI: 10.1021/tx050151l.

(4) Marx, K. A. Quartz Crystal Microbalance:  A Useful Tool for Studying Thin Polymer Films and Complex Biomolecular Systems at the Solution−Surface Interface. *Biomacromolecules* **2003**, *4* (5), 1099-1120. DOI: 10.1021/bm020116i.
